# A polygenetic risk score combined with environmental factors better predict susceptibility to hepatocellular carcinoma in Chinese population

**DOI:** 10.1002/cam4.7230

**Published:** 2024-05-02

**Authors:** Yuanlin Zou, Jicun Zhu, Caijuan Song, Tiandong Li, Keyan Wang, Jianxiang Shi, Hua Ye, Peng Wang

**Affiliations:** ^1^ Department of Epidemiology and Statistics, College of Public Health Zhengzhou University Zhengzhou Henan Province China; ^2^ Henan Key Laboratory of Tumor Epidemiology and State Key Laboratory of Esophageal Cancer Prevention & Treatment Zhengzhou University Zhengzhou Henan Province China; ^3^ Department of Pharmacy The First Affiliated Hospital of Zhengzhou University Zhengzhou Henan Province China; ^4^ The Institution for Chronic and Noncommunicable Disease Control and Prevention Zhengzhou Center for Disease Control and Prevention Zhengzhou Henan Province China; ^5^ Henan Institute of Medical and Pharmaceutical Sciences Zhengzhou University Zhengzhou Henan Province China

**Keywords:** genetic risk score, hepatocellular carcinoma, polygenic risk score, predictive models, risk factors

## Abstract

**Aims:**

This study aimed to investigate environmental factors and genetic variant loci associated with hepatocellular carcinoma (HCC) in Chinese population and construct a weighted genetic risk score (wGRS) and polygenic risk score (PRS).

**Methods:**

A case–control study was applied to confirm the single nucleotide polymorphisms (SNPs) and environmental variables linked to HCC in the Chinese population, which had been screened by meta‐analyses. wGRS and PRS were built in training sets and validation sets. Area under the curve (AUC), net reclassification improvement (NRI), integrated discrimination improvement (IDI), Akaike information criterion (AIC), and Bayesian information criterion (BIC) were applied to evaluate the performance of the models.

**Results:**

A total of 13 SNPs were included in both risk prediction models. Compared with wGRS, PRS had better accuracy and discrimination ability in predicting HCC risk. The AUC for PRS in combination with drinking history, cirrhosis, HBV infection, and family history of HCC in training sets and validation sets (AUC: 0.86, 95% CI: 0.84–0.89; AUC: 0.85, 95% CI: 0.81–0.89) increased at least 20% than the AUC for PRS alone (AUC: 0.63, 95% CI: 0.60–0.67; AUC: 0.65, 95% CI: 0.60–0.71).

**Conclusions:**

A novel model combining PRS with alcohol history, HBV infection, cirrhosis, and family history of HCC could be applied as an effective tool for risk prediction of HCC, which could discriminate at‐risk individuals for precise prevention.

## 1 INTRODUCTION

Liver cancer is one of the most common malignant tumors with high mortality.^1^ According to GLOBOCAN statistics 2020, liver cancer is the most frequent type of cancer in 11 countries and the primary cause of cancer‐related deaths in 23 countries.^2^ The burden of liver cancer in China is high: in 2020, it accounted for 45.3% of all liver cancer cases and 47.1% of deaths worldwide, with a 5‐year survival rate of only 12.1%.^3^, ^4^ Hepatocellular carcinoma (HCC), the main histologic form of primary liver cancer, accounts for roughly 90% of occurrences, and patients with advanced HCC have a median survival period of less than 1 year.^5^, ^6^ Patients with early diagnosed HCC can have a 5‐year survival rate of 70% if appropriate treatment is given, despite the fact that their prognosis is usually poor.^6^ The prerequisite for the early detection of HCC in a population is the establishment of an accurate and easy prediction model to identify individuals at different risks of developing HCC, leading to individualized and precise surveillance and prevention.^7^

Single nucleotide polymorphisms (SNPs), the most frequent type of genetic variants in humans, have been proved to contribute to HCC susceptibility.^8^ With the availability of genetic data and the lower cost of generating these data, the development of polygenic score has been facilitated. Composite polygenic scores could summarize risk variants from associated loci into a single number: either a weighted genetic risk score (wGRS) or a polygenic risk score (PRS). wGRS and PRS can make a useful contribution to identifying individuals at risk for many disorders such as colorectal cancer,^9^ liver cirrhosis,^10^ prostate cancer^11^ and lung cancer,^12^ thereby optimizing treatment and improving prognosis. For HCC, previous studies have established wGRS or PRS based on genetic variants in Western populations.^13^, ^14^ However, there is a lack of a genetic prediction model for HCC patients in Chinese population due to ethnic differences. Furthermore, PRS could be further developed in combination with nongenetic risk factors.^15^ Considering that the occurrence of HCC is the result of genetic, environmental and other factors, the combined effect of PRS and environmental factors may have utility in disease prediction.

Therefore, this study aimed to (1) screen statistically significant loci and environmental factors associated with HCC from meta‐analyses and a case–control study; (2) construct wGRS and PRS based on HCC‐related SNPs; (3) compare the predictive power of above two models; (4) establish a prediction model combined PRS and environmental factors.

## 2 METHODS AND MATERIALS

### 2.1 Meta‐analysis of risk factors for HCC

A comprehensive literature search was conducted using PubMed, Web of Science, EMBASE, Cochrane Library, CNKI (Chinese), VIP (Chinese), and Wanfang (Chinese) databases, updated as of December 31, 2020. The search phrases “China” or “Chinese”; “liver” or “hepatic” or “hepatocellular”; “cancer” or “carcinoma” or “neoplasm” or “tumor”; “risk” or “risk factor”; “single nucleotide polymorphism” or “polymorphism” or “variant” or “variation” were present in different combinations.

The inclusion criteria concerned the relationship between polymorphisms or environmental variables and HCC risk in Chinese populations, for which an odds ratio (OR) with a 95% confidence interval (CI) was available. The following were the exclusion criteria: (1) non‐Chinese subjects; (2) non‐primary HCC in the case group; (3) fewer than 10 subjects or fewer than 5 specific genotypes in cases or controls. Two researchers independently performed data extraction and quality evaluation.

### 2.2 Study population

The sample size required for this case–control study was estimated to be at least 609 individuals per group (with alpha = 0.05, power = 0.80, OR = 0.15, MAF = 0.165), calculated using PASS 15.0 software (NSCC, USA). Based on age (±5) and sex frequency matching, 633 cases and 651 controls (collected from June 2019 to September 2022) were included. The inclusion criteria for the cases were (1) age ≥18 years; (2) diagnosis of HCC; (3) no history of other malignancy; (4) without a history of gastrointestinal diseases. At the same time as cases were enrolled, controls with no history of malignant and gastrointestinal diseases and no blood relation to the cases were randomly selected from those hospitalized or undergoing physical examination. Cases and controls were recruited from a tertiary care hospital in Zhengzhou, China, and this study was authorized by the Zhengzhou University Ethics Committee (ZZURIB2019001), with all participants signing informed permission.

### 2.3 Selection SNPs and genotyping

Based on the HCC‐associated genetic variants screened through meta‐analyses, 49 SNPs in 41 genes were quantified and 26 SNPs were identified to be related to the risk of HCC. Linkage disequilibrium (LD) analysis was examined using Haploview (version 4.2). After LD analysis (*r* ^2^ > 0.8), a total of 24 candidate SNPs were retained.

The sample DNA was extracted using the DNA Extraction Kit (Changzhou GenMagBio Biotechnology Co. LTD), and candidate SNPs were genotyped by Improved Multiplex Ligation Detection Reaction (iMLDR™). To ensure that the SNP typing results were correct, 5% of the samples were chosen at random to validate their sequencing results.

### 2.4 wGRS and PRS

The average population risk (genetic score) for each of the 13 SNPs screened by meta‐analyses and confirmed by a case–control study was calculated using the genotype frequency of the heritable variation (HapMap CHB population data) and pooled OR of the meta‐analyses.

$$\begin{array}{c} \text{Genetic score}\;\left(W\right)={\left(1-P\right)}^{2}+2P\left(1-P\right)\text{OR}\\ \;+P^{2}{\text{OR}}^{2},P\;\text{is the risk allele frequency}. \end{array}$$

Assuming that the genotypes of a SNP are AA, AB, and BB, with *A* being the non‐risk allele and *B* being the risk allele, and the corresponding risk values are 1, OR, and OR^2^, respectively. The following is an estimate of the wGRS.

$$\mathrm{AA}=1/W;\mathrm{AB}=\text{OR}/W;\mathrm{BB}={\text{OR}}^{2}/W.$$

$$\text{wGRS}={\mathrm{SNP}}_{1}\times {\mathrm{SNP}}_{2}\times {\mathrm{SNP}}_{3}\ldots \ldots {\mathrm{SNP}}_{11}\times {\mathrm{SNP}}_{12}\times {\mathrm{SNP}}_{13}.$$

PRS aims to quantify the combined effect of various loci by converting information about numerous genetic variants associated with attributes into scores that indicate an individual's susceptibility to disease.

PRS = $\sum_{i}^{m}\beta_{i}\left(\sum_{j=0}^{2}\omega_{\mathrm{ij}}\times j\right)$; *m* is the total number of disease‐associated SNPs, *i* is the serial number of the SNPs, β_*i*_ is the effect size of the *i*th SNP, and ω_*ij*_ is the probability of observing genotype *j*.

The wGRS and PRS were categorized by quintiles into low‐risk group (quintile 1 [Q1], 0%–20%), medium‐risk group (Q2–4, 21%–80%), and high‐risk group (Q5, 81%–100%).

### 2.5 Statistical analysis

*Q* test and *I* ^2^ test were performed to evaluate the heterogeneity of the studies included in meta‐analyses. If *p* > 0.05 or *I* ^2^ < 50%, there was no heterogeneity and the fixed‐effects model was performed to integrate the data; otherwise, there was heterogeneity and the random‐effects model was selected to merge the data. Sensitivity analysis was conducted by excluding one study at a time to explore potential heterogeneity and evaluate the stability of the pooled results. Egger's and Begger's tests were applied to assess publication bias. To assess the reliability of the statistically significant association, the false positive reporting probability (FPRP) test and the Venice criteria were calculated.

For continuous variables, data with normal distribution were presented as mean ± standard deviation and analyzed by Student's *t*‐test. For categorical variables, data were shown as actual numbers and percentages and compared using Pearson's chi‐squared test. A chi‐squared goodness‐of‐fit test was used to determine the Hardy–Weinberg equilibrium (HWE) of the genotype distribution in the controls. The relationship between SNPs and HCC susceptibility was evaluated using unconditional logistic regression analysis with adjustment for drinking history, T2DM, HBV infection, cirrhosis, and family history of HCC.

Quality control of the genetic data, association analysis of the allele and generation basic dataset and target dataset of PRSice‐2 (Gavin Band, New York, USA) were performed using Plink 1.9 program (NIH‐NIDDK's Laboratory of Biological Modeling, Harvard University). The predictive ability of different models was contrasted by receiver operating characteristic (ROC) and area under the curve (AUC). The statistically significant differences in the AUC were calculated using the DeLong test. The predictive degree of different models was estimated using net reclassification improvement (NRI) and integrated discrimination improvement (IDI). Model fit was checked using the Akaike information criterion (AIC) and the Bayesian information criterion (BIC). Sensitivity analyses were performed to test the robustness of the model by separately excluding patients who were not infected with HBV or non‐cirrhotic liver.

All statistical analysis was performed by R software (version 4.2.2), Stata (version 17.0), and SPSS (version 21.0). A two‐tailed *p* < 0.05 was regarded as statistically significant.

## 3 RESULTS

### 3.1 Meta‐analysis of risk factors for HCC

**FIGURE 1:**
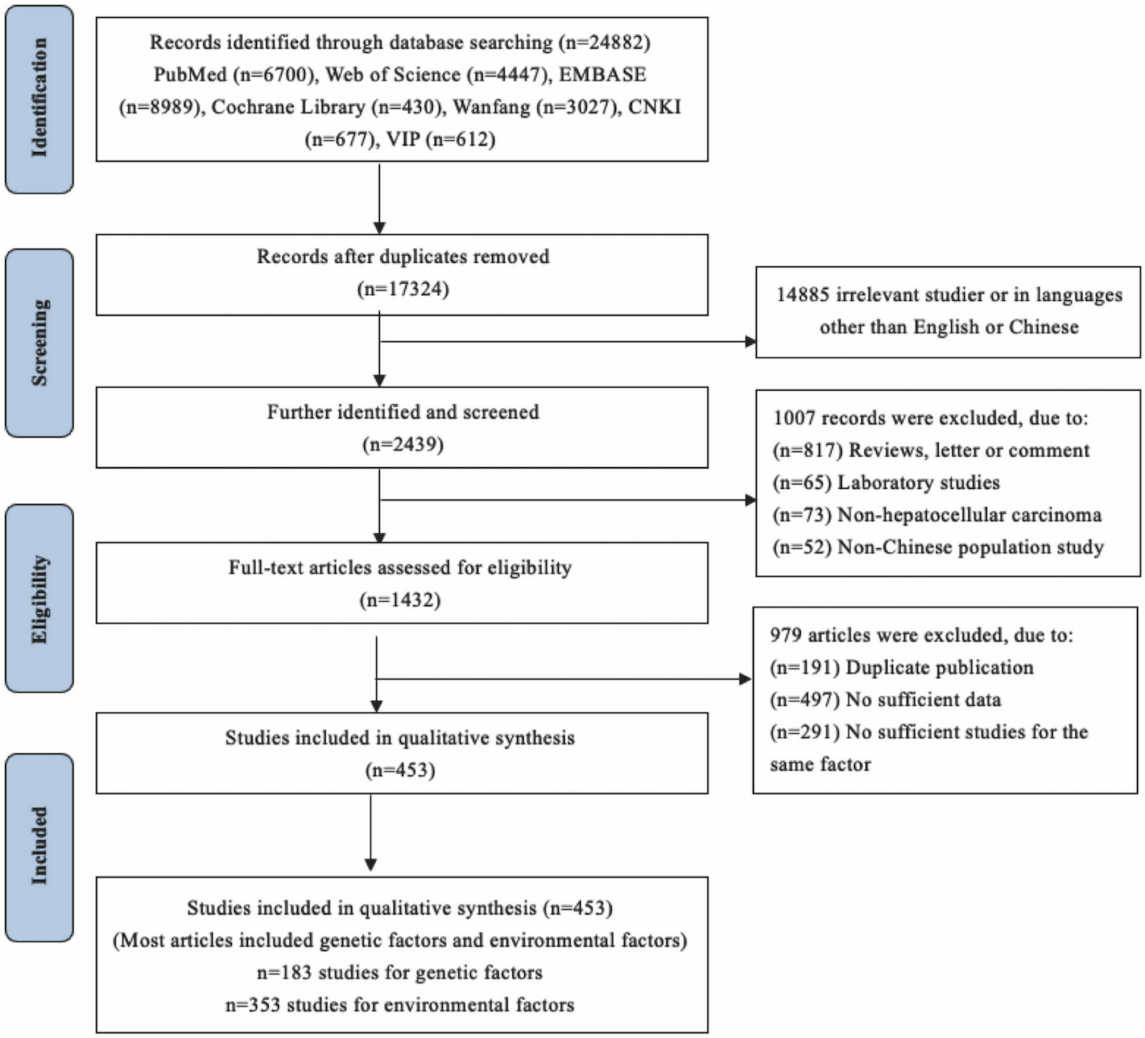
Flow chart of literature search and study selection.

Figure 1 depicted a flow diagram illustrating the literature search technique. A total of 453 articles (353 studies for environmental factors; 183 studies for genetic factors) were chosen for quantitative synthesis based on the search technique (Supplemental references). The meta‐analyses of environmental and genetic risk factors for HCC are shown in Tables S1–S3. HBV infection, HCV infection, smoking, drinking, fatty liver, cirrhosis, family history of HBV, HCC and tumors, T2DM, aflatoxin‐contaminated food, fried, and smoked foods were statistically significantly associated with HCC (*p* < 0.05) (Table S1). Meanwhile, 26 SNPs, such as *IL‐6* rs1800796, *COX‐2* rs5275/rs689466, *IL‐8* rs4073, were associated with genetic susceptibility to HCC (*p* < 0.05) (Table S2).

Sensitivity analysis revealed that the overall pooled ORs were not influenced by any individual study, indicating the pooled ORs were still stable (Data not shown). The results of Begger's and Egger's tests demonstrated that there was no potential publication bias for most factors (Table S3).

According to a priori probabilities of 0.15, 0.1, and 0.01, there were 26, 25, and 12 SNPs with FPRP<0.5 and the Venice criterion showed a high level of evidence for rs1801133 (AAA) (Table S4).

### 3.2 Basic characteristics of study subjects

In Table 1, the basic information of 633 HCC patients and 651 controls from a case–control study were presented. There were 530 (87.73%) males and 103 (16.27%) females in the case group with an average age of 50.57 years, and the control group included 519 (79.72%) males and 132 (20.28%) females with an average age of 49.60 years. In the case group, the proportions of drinking alcohol, HBV infection, cirrhosis, family history of HCC, and T2DM were higher than those in the control group (*p* < 0.05). Multivariable logistic regression analysis indicated that drinking history, HBV infection, liver cirrhosis and family history of HCC were significantly related to the risk of HCC (Table 1).

**TABLE 1:** Basic characteristics of 633 HCC patients and 651 controls.

Factors	Cases (*N* = 633)	Control (*N* = 651)	*p*	OR (95% CI)^a^	OR (95% CI)^b^
Age	50.57 ± 8.27	49.60 ± 12.29	0.098	1.01 (1.00, 1.02)	
Sex					
Man	530 (83.73)	519 (79.72)	0.064	1	
Woman	103 (16.27)	132 (20.28)		0.76 (0.58, 1.06)	
Smoking history					
No	397 (62.72)	425 (65.28)	0.338	1	
Yes	236 (37.28)	226 (34.72)		1.12 (0.89, 1.40)	
Drinking history					
No	336 (53.08)	417 (64.06)	0.003	1	1
Yes	297 (46.92)	234 (35.94)		1.40 (1.12, 1.76)*	1.80 (1.35, 2.41)*
Family history of HCC					
No	571 (90.21)	635 (97.54)	<0.001	1	1
Yes	62 (9.79)	16 (2.46)		4.31 (2.46, 7.55)*	3.38 (1.72, 6.66)*
HBV infection					
No	145 (22.91)	382 (58.68)	<0.001	1	1
Yes	488 (77.09)	269 (41.32)		4.70 (3.69, 5.98)*	1.79 (1.32, 2.43)*
Cirrhosis					
No	135 (21.33)	525 (80.65)	<0.001	1	1
Yes	498 (78.67)	126 (19.35)		4.78 (3.75, 6.09)*	12.06 (8.92, 16.29)*
T2DM					
No	544 (85.94)	601 (92.32)	<0.001	1	1
Yes	89 (14.06)	50 (7.68)		1.97 (1.37, 2.83)*	1.31 (0.84, 2.06)

Abbreviations: CI, confidence intervals; HBV, hepatitis B virus; HCC, hepatocellular carcinoma; OR, odd ratios; T2DM, type 2 diabetes mellitus.

* *p* < 0.05.

^a^ Unadjusted analysis.

^b^ Adjusted for drinking history, HBV infection, cirrhosis, family history of HCC, and T2DM.

### 3.3 Association of candidate SNPs with genetic susceptibility to HCC

Two of the 26 SNPs were excluded due to LD. The distribution of the remaining 24 SNP genotypes in the control group conformed to the HWE test. After adjusting for drinking history, T2DM, HBV infection, cirrhosis, and family history of HCC, genetic association analysis of 24 candidate SNPs by multivariate logistic regression showed that 13 SNPs (rs689466, rs1800872, rs1799964, rs2228001, rs2279744, rs1042522, rs1801133, rs1800566, rs738409, rs7574865, rs2910164, rs11614913, rs3746444) were associated with genetic susceptibility to HCC (*p* < 0.05) (Figures S1–S4).

### 3.4 Construction of wGRS and PRS

**FIGURE 2:**
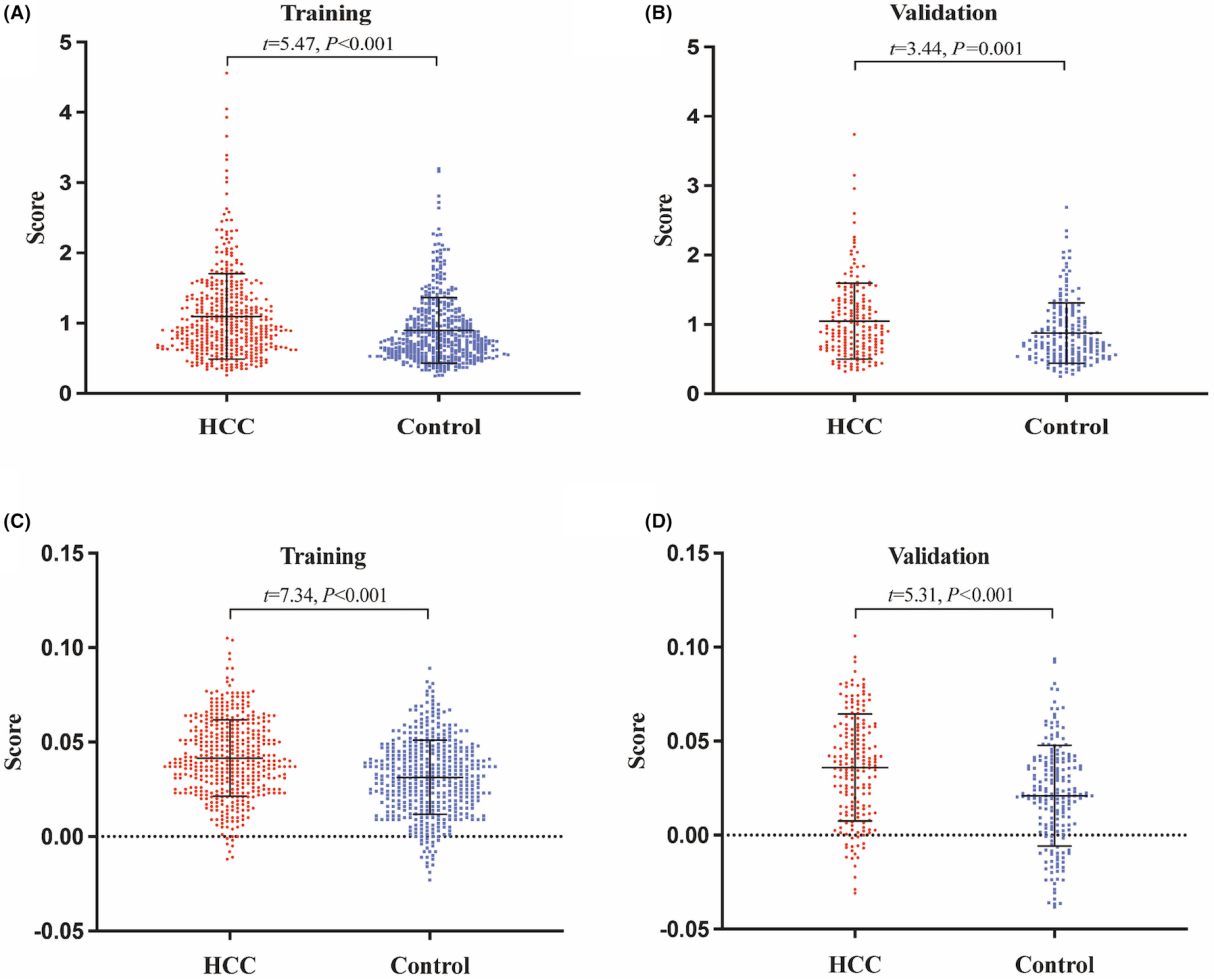
Distribution of wGRS and PRS in HCC and control. (A) wGRS in training set; (B) wGRS in validation set; (C) PRS in training set; (D) PRS in validation set.

**FIGURE 3:**
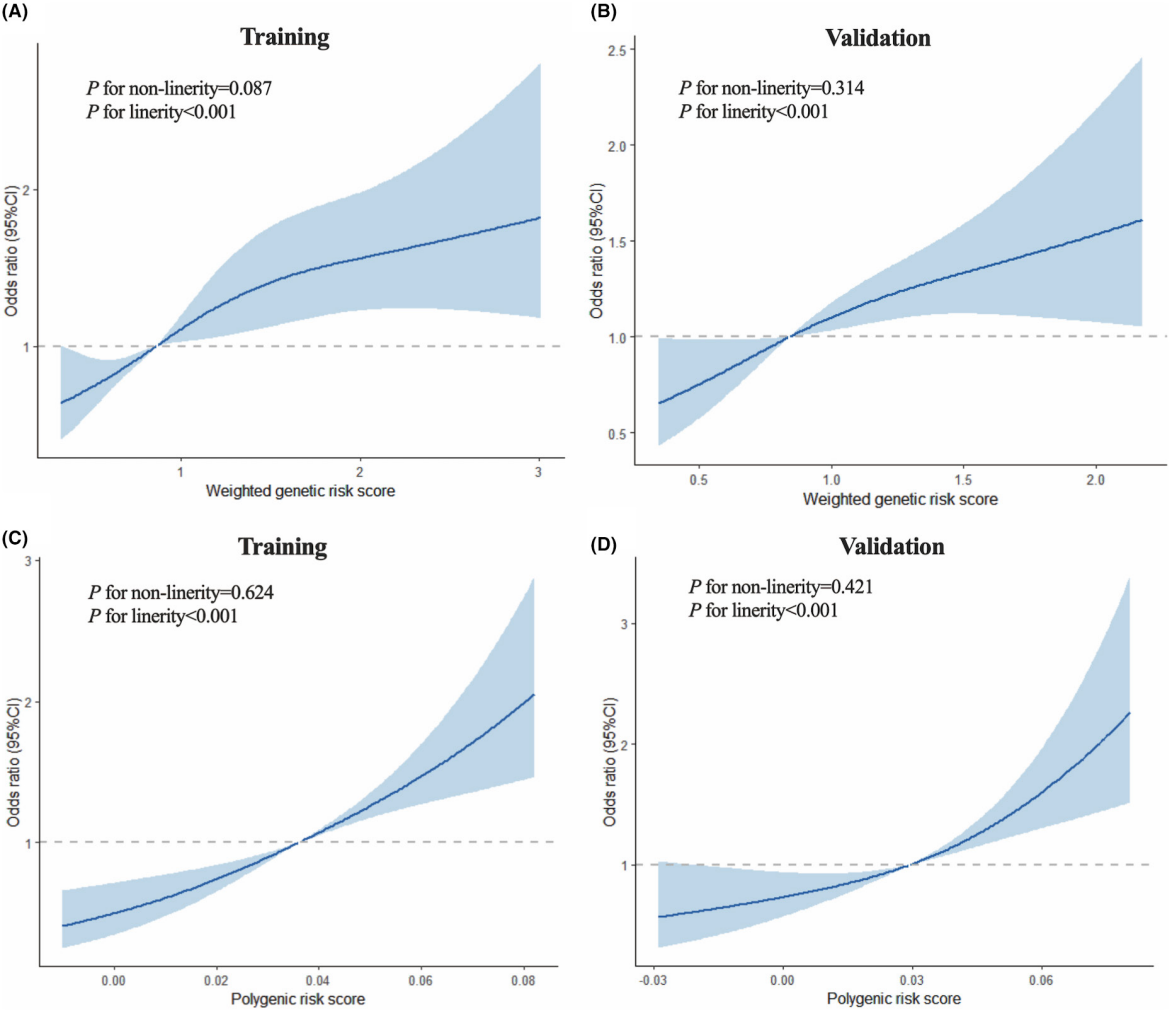
Dose–response relationship curves of wGRS and PRS with the risk of HCC. (A) wGRS in training set; (B) wGRS in validation set; (C) PRS in training set; (D) PRS in validation set.

All subjects were randomly assigned to the training set and the validation set in a ratio of 7:3. The average values of the two genetic risk‐scoring models based on wGRS and PRS were higher in the HCC group than in the control group in both the training and validation sets (*p* < 0.05) (Figure 2). The restricted cubic spline curves displayed linear and positive relationships between the values of the two models and HCC risk (*p* < 0.05) (Figure 3). Individuals in the high genetic risk group exhibited an elevated risk of HCC in both prediction models when compared to those in the low genetic risk group (*p* < 0.05) (Table 2).

**TABLE 2:** Regression analysis of subgroups of PRS and GRS and risk of HCC.

Group	HCC		Control		OR (95% CI)	*p*
	*n* (%)	$\bar{x}$ ± *s*	*n* (%)	$\bar{x}$ ± *s*		
wGRS						
Training set						
Low genetic risk	64 (14.45)	0.45 ± 0.07	116 (25.44)	0.46 ± 0.08	1	‐
Median genetic risk	265 (59.82)	0.97 ± 0.21	274 (60.09)	0.87 ± 0.21	1.75 (1.24, 2.49)	0.002
High genetic risk	114 (25.73)	1.89 ± 0.62	66 (14.47)	1.80 ± 0.42	3.13 (2.04, 4.81)	<0.001
Validation set						
Low genetic risk	28 (14.74)	0.44 ± 0.06	49 (25.13)	0.45 ± 0.08	1	‐
Median genetic risk	113 (59.47)	0.89 ± 0.21	118 (60.51)	0.85 ± 0.21	1.68 (0.99, 2.85)	0.057
High genetic risk	49 (25.79)	1.78 ± 0.51	28 (14.36)	1.71 ± 0.34	3.06 (1.59, 5.91)	0.001
PRS						
Training set						
Low genetic risk	55 (12.42)	0.009 ± 0.008	125 (27.41)	0.006 ± 0.010	1	‐
Median genetic risk	265 (59.82)	0.032 ± 0.010	274 (60.09)	0.031 ± 0.010	2.20 (1.53, 3.15)	<0.001
High genetic risk	123 (27.77)	0.060 ± 0.011	57 (12.50)	0.059 ± 0.010	4.90 (3.14, 7.66)	<0.001
Validation set						
Low genetic risk	28 (14.74)	−0.009 ± 0.015	49 (25.13)	−0.014 ± 0.013	1	‐
Median genetic risk	106 (55.79)	0.030 ± 0.014	126 (64.62)	0.027 ± 0.012	1.46 (0.86, 2.48)	0.154
High genetic risk	56 (29.47)	0.069 ± 0.012	20 (10.26)	0.067 ± 0.012	4.99 (2.50, 9.94)	<0.001

Abbreviations: CI, confidence intervals; HCC, hepatocellular carcinoma; OR, odd ratios; PRS, polygenic risk score; wGRS, weighted genetic risk scores.

**FIGURE 4:**
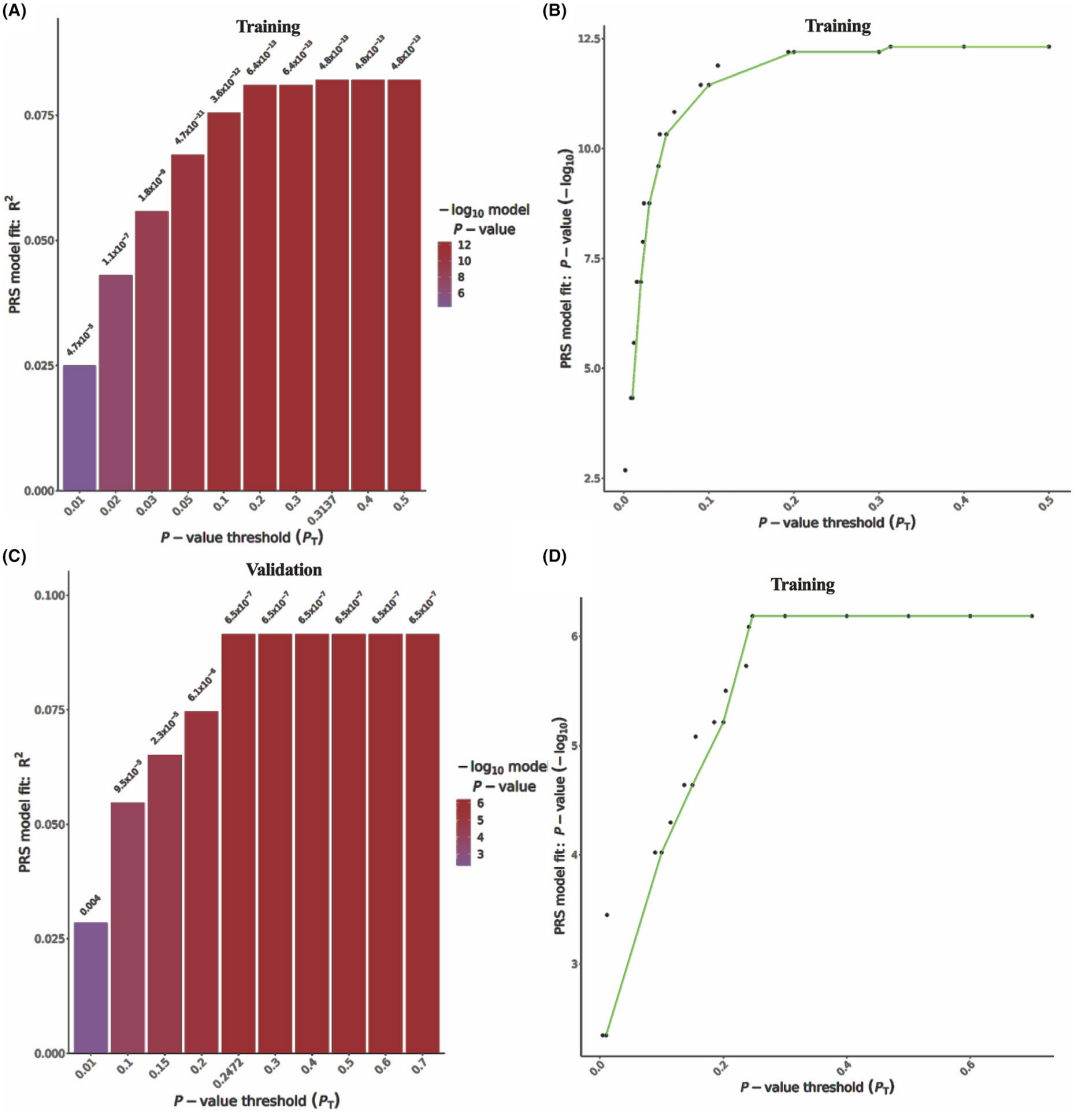
The model fit of the PRS and high‐resolution plots. (A) PRS prediction threshold of HCC and phenotypic variation interpretation bar plot in training set and (C) validation set; (B) PRS prediction threshold and model goodness of fit high‐resolution plot in training set and (D) validation set. HCC, hepatocellular carcinoma; PRS, polygenic risk score.

The bar plot for the PRS model indicated the variance ratio of the correlation findings obtained at various *p*‐value thresholds (*p*_t_), which was the distribution of the explained values (*R* ^2^) of the estimated phenotypic variance (Figure 4). In the training set, the highest point in the column graph represented the best model (*p*_t_ = 0.314), and genetic variation accounted for approximately 8.2% of the cases (*p* = 4.8 × 10^−13^). Meanwhile, in the validation set, the point in the column histogram was highest at *p*_t_ = 0.247, and the genetic variation could explain 9.2% of the cases (*p* = 6.5 × 10^−7^). In addition, the same results were also observed in the output results of PRSice‐2, which displayed the empirical *p*‐value distribution corresponding to the relation results acquired under varied *p*_t_ values with the outcomes of high‐resolution plots.

### 3.5 Evaluation of wGRS and PRS

The calibration curves of both models exhibited good agreement between the predicted and actual probabilities for both training and validation sets (Figure S5). The difference in AUC between PRS and wGRS was not significant in both the training set and the validation set (Delong *p* > 0.05). PRS outperformed wGRS by the values of AIC and BIC (Table 3). Besides, the results of NRI and IDI demonstrated that the PRS model was superior to the wGRS model in accuracy and discrimination capability. Therefore, PRS was selected to create a new model along with environmental factors to improve the model's prediction capabilities.

**TABLE 3:** Comparison of AUC, AIC, BIC, NRI, and IDI between different risk prediction model.

Model	AUC (95% CI)	Delong *p*	AIC	BIC	NRI (95% CI)	IDI (95% CI)
Training set						
wGRS	0.61 (0.57, 0.65)	‐	1414.04	1233.64	‐	‐
PRS	0.63 (0.60, 0.67)	0.122^a^	1197.66	1207.26	0.21 (0.14, 0.28)^a^, *	0.03 (0.02, 0.04)^a^, *
PRS + environmental factors	0.86 (0.84, 0.89)	<0.001^b^	842.79	871.60	0.78 (0.68, 0.87)^b^, *	0.35 (0.32, 0.38)^b^, *
Validation set						
wGRS	0.60 (0.54, 0.66)	‐	525.66	533.57	‐	‐
PRS	0.65 (0.60, 0.71)	0.068^a^	506.71	514.61	0.22 (0.09, 0.35)^a^, *	0.04 (0.02, 0.06)^b^, *
PRS + environmental factors	0.85 (0.81, 0.89)	<0.001^b^	381.10	404.82	0.69 (0.54, 0.84)^b^, *	0.31 (0.27, 0.36)^b^, *

Abbreviations: AIC, Akaike information criterion; AUC, area under curve; BIC, Bayesian information criterion; CI, confidence intervals; IDI, integrated discrimination improvement; NRI, net reclassification improvement; PRS, polygenic risk score.

* *p* < 0.05.

^a^ PRS versus wGRS.

^b^ PRS + environmental factors versus PRS; environmental factors: drinking history, HBV infection, cirrhosis and family history of HCC.

**FIGURE 5:**
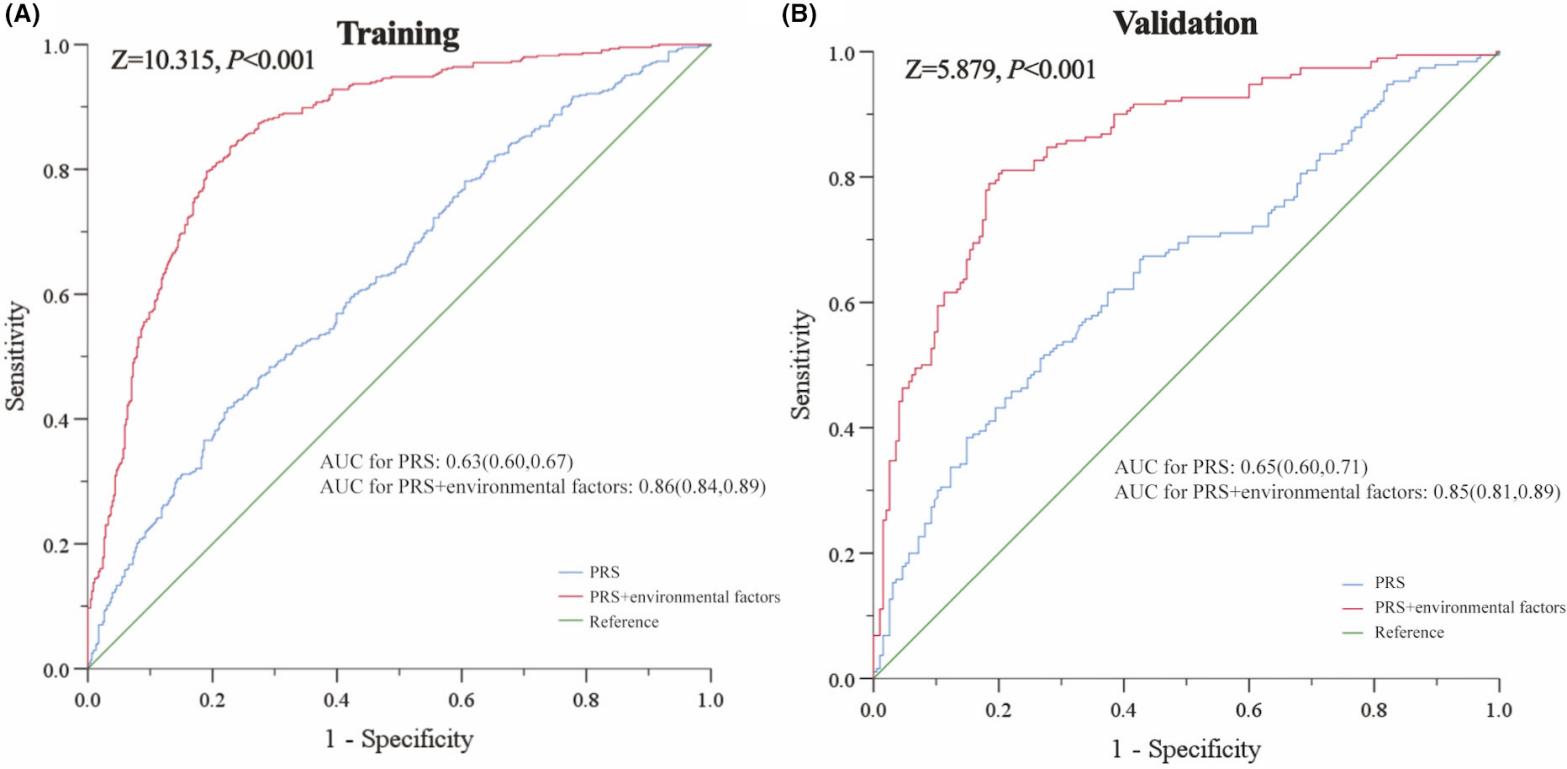
ROC curves of prediction models for risk of HCC. AUC, area under curve; HCC, hepatocellular carcinoma; PRS, polygenic risk score; ROC, receiver operating characteristic.

After constructing the multivariate regression model by combining PRS with the four environmental factors, the AUC of the comprehensive model performed significantly better in both training and validation sets (AUC for training set 0.86, 95% CI: 0.84–0.89; AUC for validation set 0.85, 95% CI: 0.81–0.89) (Delong *p* < 0.05) (Table 3; Figure 5). Furthermore, the accuracy of reclassification and the ability of comprehensive discrimination of both training and validation sets were improved (Table 3).

### 3.6 Sensitivity analysis of HCC risk prediction models

The sensitivity analysis was conducted by excluding patients without HBV infection or cirrhosis (Table S5). The AUC of PRS plus environmental factors model was significantly higher than the model of the PRS alone in patients with HBV infection (Delong *p* < 0.001). Besides, in the HBV‐infected or cirrhotic group, the addition of environmental factors to the model decreased both AIC and BIC and improved the predictive ability as measured by IDI (*p* < 0.001) and NRI (*p* < 0.001).

## 4 DISCUSSION

In the present study, drinking history, HBV infection, cirrhosis, and family history of HCC were identified as risk factors for HCC after multivariable adjustment. Based on meta‐analyses screening and association analysis of a case–control study, a total of 13 SNPs were found to be associated with HCC susceptibility. In addition, wGRS and PRS were constructed based on these SNPs to evaluate the potential clinical benefit. In both models, individuals with high genetic risk scores were more likely to develop HCC than those with low genetic risk scores. In comparison to wGRS, the PRS enabled great accuracy and discrimination ability in predicting HCC cases, and the AUC increased by at least 20% when the PRS was combined with alcohol history, HBV infection, cirrhosis, and family history of HCC. Thus, our study is critical for the early detection of HCC in high‐risk populations.

Currently, most scholars believe that many factors cause HCC, and the major ones are environmental factors and genetic factors.^16^, ^17^ Geographically, chronic infection with HBV predominated as the cause of HCC in Asia, particularly in developing countries.^18^ Consistent with previous findings, our study also observed that HBV significantly enhanced the risk of HCC. A previous cohort study conducted in Taiwan showed that vaccination of infants reduced the incidence of HCC from 0.92 to 0.23/10^5^ person‐years.^19^ Therefore, HBV vaccination is a cost‐effective and reliable method to reduce HBV infections.^20^ Nevertheless, if cirrhosis was diagnosed, there was a risk of HCC even after successful antiviral therapy.^21^ The results of our study indicated that cirrhosis can increase the risk of HCC by more than tenfold. A published study has shown that 80%–90% of new cases of HCC occur in association with liver cirrhosis.^22^ A meta‐analysis further demonstrated that patients with primary biliary cirrhosis had an 18‐fold higher risk of HCC than the general population.^23^ Parallel cohort studies conducted in both the United States and China among HCV patients yielded similar results.^24^ Notably, the latest report from the National Cancer Center of China in 2022 reported an incidence rate of only 15.05 per 100,000 for liver cancer.^25^ However, an 11‐year retrospective study conducted in China identified that 4.13% of patients with primary biliary cirrhosis developed HCC^26^ while a UK cohort study found the cumulative 10‐year incidence of HCC among patients with cirrhosis caused by HCV was 4%.^27^ Collectively, these studies underscore the heightened vulnerability of cirrhotic patients to HCC development. Nevertheless, a new insight has emerged that cirrhosis may be a reaction to govern tissue regeneration and clonal growth rather represented a predisposition to HCC development.^28^ A study suggested that fibrotic septa encircle the microscopic distribution of regenerating nodules in cirrhotic liver, potentially spatially limiting the area available for tumor clones, and preventing the spread of cancer cells.^29^ Moreover, fibrosis development and cirrhosis‐induced inflammation might prime the immune system, leading to a superior reaction to liver cancer cells.^28^ Consequently, further investigations should focus on the unique involvement of fibrosis and the role of the immune response during the development of HCC.

SNPs, common genetic factors, could affect susceptibility to HCC. Our study has identified 13 SNPs with an association with HCC. These SNPs were categorized according to the functions of the genes in which they are located: inflammation and immune response (*COX‐2* rs689466, *IL‐10* rs1800872, *TNF‐α* rs1799964), DNA synthesis and damage repair (*XPC* rs2228001, *MDM2* rs2279744, *TP53* rs1042522), pathways of metabolic (*MTHFR* rs1801133, *NQO1* rs1800566, *PNPLA3* rs738409), and signaling (*STAT4* rs7574865, *miR‐146a* rs2910164, *miR‐196a2* rs11614913, *miR‐499* rs3746444). Two widely studied SNPs were *PNPLA3* rs738409 and *miR‐196a2* rs11614913. The *PNPLA3* rs738409 C > G, contributing to hepatic fat accumulation and liver damage, may be associated with HCC development.^30^, ^31^ Gene homeobox (HOX) and annexin A1 (ANXA1) are the targets of *miR‐196a2* and play crucial roles in carcinogenesis and malignant transformation of HCC.^32^, ^33^, ^34^ The variation of *miR‐196a2* rs11614913 C > T not only affects the transcriptional level of mature miR‐196a, but also has a biological effect on the production of target genes.^35^

Since HCC is a polygenic illness, a single gene mutation is not representative for evaluating the risk of HCC. wGRS and PRS are commonly used to combine information across loci.^36^, ^37^ A cohort study in UK concluded that PRS could improve diagnostic accuracy and positive predictive values for severe liver disease in risk classes with moderate to high clinical scores.^38^ Gellert‐Kristensen et al.^39^ constructed a wGRS model utilizing three genetic variations in a European population and the results showed that the individuals in the high genetic risk category (Scores 5–6) were 29 times more likely to develop HCC. Nahon et al.^14^ discovered that a 7‐SNPs wGRS could be an independent risk factor to predict 5‐year HCC incidence. However, the population of these studies was from Europe and it is unclear whether the study is suitable for other populations given the ethnic differences. Besides, few studies have compared the predictive performance of HCC between wGRS and PRS models. In this study with Chinese as the subject, wGRS and PRS were applied to evaluate the overall contribution of 13 SNP gene variants subjected to two‐stage selecting, and the results displayed that a high risk score assessed by estimating wGRS or PRS had at least a twofold increased risk of HCC. Moreover, PRS had better prediction capacity than wGRS, which was close to the lately published study.^40^

Based on the results of ROC curve and AUC, a new model incorporating PRS with environmental factors could significantly boost the prediction ability. In line with our results, Duan et al.^40^ confirmed that model predicting the risk of gastric cancer could be optimized by combining PRS and behavioral factors. Similar effects of PRS combined with environmental variables have also been found in breast and lung cancer.^12^, ^41^

Several limitations must be considered. First, there may be publication bias in meta‐analyses, meaning that statistically significant results are more likely to be reported. Second, our study assessed 26 SNPs associated with HCC risk in the Chinese population through a case–control study, which may restrict the generalization of our findings to other ethnic groups with varying allele frequencies, LD patterns, and variant impact sizes. Third, the information collection was not detail enough such as the lack of the frequency of smoking and drinking to enable subgroup analysis. In addition, the potential interactions between genetic variations and environmental variables were not taken into account. In the future studies of genetic risk scores model, rare structural genetic alterations, copy number variants, and noncoding variants should be considered.

## 5 CONCLUSION

In conclusion, the PRS had better predictive ability for HCC than the wGRS. The PRS combined with drinking history, HBV infection, cirrhosis, and family history of HCC had a high accuracy of fitting and prediction effect, which is conducive to early warning and accurate screening of HCC high‐risk groups.

## AUTHOR CONTRIBUTIONS

**Yuanlin Zou:** Data curation (equal); formal analysis (equal); methodology (equal); software (equal); writing – original draft (equal); writing – review and editing (equal). **Jicun Zhu:** Data curation (equal); formal analysis (equal); methodology (equal); software (equal); writing – original draft (equal); writing – review and editing (equal). **Caijuan Song:** Data curation (equal); writing – review and editing (equal). **Tiandong Li:** Data curation (equal); writing – review and editing (equal). **Keyan Wang:** Data curation (equal); writing – review and editing (equal). **Jianxiang Shi:** Data curation (equal); writing – review and editing (equal). **Hua Ye:** Data curation (equal); writing – review and editing (equal). **Peng Wang:** Conceptualization (lead); supervision (lead); writing – review and editing (equal).

## FUNDING INFORMATION

This work was supported by the Funded Project of the Zhengzhou Major Project for Collaborative Innovation (18XTZX12007) and the National Science and Technology Major Project of China (2018ZX10302205).

## CONFLICT OF INTEREST STATEMENT

The authors declare no conflict of interest for this article.

## ETHICS STATEMENT

All subjects provided informed consent, and the Ethics Committee of Zhengzhou University authorized this study (ZZURIB2019001).

## Supporting information


Data S1.

Data S2.

## Data Availability

The data that support the findings of this study are available from the corresponding author upon reasonable request.
