# Supplementary material for: A polygenetic risk score combined with environmental factors better predict susceptibility to hepatocellular carcinoma in Chinese population
Source: Cancer Med. 2024 May 2;13(9):e7230. doi: 10.1002/cam4.7230 (PMC11066500; doi:10.1002/cam4.7230)
Supplement: Supplementary file 1 — Data S1. [file CAM4-13-e7230-s001.docx]

Reference

[1] Ji SW, Wang JB. The risk analysis of type 2 diabetes mellitus to the development of primary liver carcinoma[J]. Chinese Hepatology, 2007,12(3): 168-170.

[2] Xing SL. Association of type 2 diabetes with hepatocellular carcinoma with chronic hepatitis B[D]. Shanxi: Shanxi Medical University, 2016.

[3] Su HY, He M, Jia ZF, et al. Etiological risk analysis of 224 cases with primary hepatic carcinoma[J]. Modem Preventive Medicine, 2008, 35(11): 2149-2151.

[4] Wang MH. The single nucleotide polymorphism (rs4963) in ADD1 is associated with susceptibility to gastric cancer and liver cancer[D]. Beijing: Peking Union Medical College, 2013.

[5] Lu HX. A case-control study on the correlation between hepatocellular carcinoma and polymorphisms of CYP3A5 and CYP3A7 genes in an area of high AFB1 contamination[D]. Guangxi: Guangxi Medical University, 2007.

[6] Tian L. Case-control study of primary hepatic carcinoma and AKT1, MDM2 gene polymorphism[D]. Xinjiang: Xinjiang Medical University, 2016.

[7] Ye XP, Peng T, Liu TW, et al. Association between aldehyde dehydrogenase-2/cytochrome P450 2E1 genetic polymorphism and habit of alcohol drinking and the susceptibility of hepatocellular carcinoma [J]. Journal of Hygiene Research, 2010, 39(01): 42-45.

[8] Zhang TW. Copy number variation of APOBEC3B is associated with susceptibility to HBV-related hepatocellular carcinoma[D]. Beijing: Peking Union Medical College, 2011.

[9] Wang CP, Su CH, Lin Y, et al. CCND1 gene polymorphism and susceptibility to hepatocellular carcinoma: a case control study[J]. Modern Preventive Medicine, 2017, 44(03): 553-556.

[10] Guo FF. Correlation analysis of CD147 functional SNP with susceptibility to primary hepatocellular carcinoma and study of its biological effects[D]. Guangxi: Guangxi Medical University, 2018.

[11] Fan XJ, Qiu XQ, Yu HP, et al. Association of COX-2 gene SNPs with the risk of hepatocellular carcinoma[J]. Chinese Journal of Cancer Prevention and Treatment, 2011, 18(06): 405-409.

[12] Xu DK, Zhang XM, Zhao P, et al. Association between single nucleotide polymorphisms in promoter of COX-2 gene and hereditary susceptibility to hepatocellular carcinoma [J]. Chinese Journal of Hepatobiliary Surgery, 2008, 14(12): 840-843.

[13] Shao WW, Fu ZZ, Wang GX, et al. Association of COX-2 8473T＞C genetic variant and risk of primary hepatic carcinoma [J]. Journal of Hebei United University (Health Sciences), 2014, (2): 141-142.

[14] Yuan GH, Dai C, Wu ZP, et al. Relationship between polymorphism of cyclinA2 gene and susceptibility to Hepatocellular carcinoma[J]. Medical Information, 2018, 31(22): 77-80.

[15] Zhu WC, Chen Q, Luo CL, et al. Relationship Study Between Gene Polymorphism of CYP1A1, GSTM1 and Genetic Susceptibility of Primary Hepatocellular Carcinoma[J]. Chinese Journal of Cancer Prevention and Treatment, 2001, 8(6): 572-574.

[16] Di J. Qinghai Tibetan region of CYP2E1 gene polymorphism and susceptibility to hepatocellular carcinoma[D]. Qinghai: Qinghai University, 2012.

[17] Liu JL, Pu H, Ma LT, et al. Risk factors analysis and a new risk scoring system predicting hepatocarcinogenesis for chronic genotype C HBV infected patients [J]. Chinese Journal of Epidemiology, 2018, 39(11): 1459-1464.

[18] Ren AH. The DDX39 protein expression and relationship of DDX39 single nucleotide polymorphism and genetic susceptibility to hepatocellular carcinoma in clusters of families with liver cancer[D]. Guangxi: Guangxi Medical University, 2017.

[19] Liu CX. DEPDC5 genetic polymorphism is not associated with susceptibility to hepatocellular carcinoma in Northern Chinese patients with chronic hepatitis B[D]. Shandong: Shandong University, 2014.

[20] Wang Z. The role of single nucleotide polymorphism of DICER gene and its expression in the occurrence and development of hepatocellular carcinoma[D]. Henan: Zhengzhou University, 2016.

[21] Liu Y. The relationship between single-nucleotide polymorphism of DNA mismatch repair gene and primary hepatocellular carcinoma[D]. Hebei: Hebei medical university, 2015.

[22] Wang QQ. Association between miRNA-binding site polymorphisms in DNA repair gene RAD52 3'-UTR and hepatocellular carcinoma[D]. Guangxi: Medical College of Guilin, 2017.

[23] Li LM. Association of DNA repair gene XPC and XPG polymorphism with genetic susceptibility to hepatocellular carcinoma[D]. Guangxi: Guangxi Medical University, 2010.

[24] Zeng XY, Qiu XQ, Ji L, et al. Study on the relationship between hepatocellular carcinoma and the interaction between polymorphisms in DNA repair gene XPD and environmental factors [J]. Chinese Journal of Epidemiology, 2009, (07): 702-705.

[25] Zhang H. Polymorphism of the DNA repair genes hOGG1 and MBD4 in Chinese population and their association with risk of hepatocellular carcinoma[D]. Henan: Zhengzhou University, 2003.

[26] Xu L. A case-control study of DNA repair gene polymorphisms and susceptibility to primary liver cancer[D]. Shanghai: Fudan University, 2003.

[27] Huang YL. The relationship between ERCC1 and AFP single nucleotide polymorphisms and hepatocellular carcinoma[D]. Guangxi: Guangxi Medical University, 2019.

[28] Li YQ. A study of the relationship between single nucleotide polymorphisms of ERCC1, TNF-α and the susceptibility to hepatocellular carcinoma in Guangxi Zhuang population[D]. Guangxi: Guangxi Medical University, 2017.

[29] Xu GH, Xu ZQ, He J, et al. Relationship between ERα-29 polymorphisms and primary liver cancer associated with HBV infection [J]. Chinese Journal of Gerontology, 2018, 38(2): 371-373.

[30] Niu Y, Li XF, Hou XF, et al. Relationship of ERα-29 Gene Polymorphisms and HBV-related Hepatocellular Carcinoma [J]. Progress in Modern Biomedicine, 2015, 15(32): 6245-6247.

[31] Yang WG, Li YH, Xu H, et al. The Relationship between ERα-29 Gene Polymorphisms and HBV-related Hepatocellular Carcinoma[J]. Progress in Modern Biomedicine, 2016, 16(30): 5908-5910+5903.

[32] Wang HL, Tang YH, Ding Z, et al. Association of Fas/FasL gene polymorphisms with susceptibility to hepatocellular carcinoma[J]. Chinese Clinical Oncology, 2017, 22(10): 913-918.

[33] Huang XL. Association between Fas gene polymorphisms and susceptibility HBV-related liver diseases[D]. Guangxi: Guangxi Medical University, 2015.

[34] Yang Y. Association of fibroblast growth factor receptor 4 genetic polymorphism with hepatocellular carcinoma susceptibility[D]. Shanghai: Second Military Medical University, 2008.

[35] Li ZQ, Huang WW, Lin ZB, et al. Genetic association analysis of the rs455804 variant in GRIK1 and the risk of liver cancer [J]. Journal of Guangdong Pharmaceutical University, 2017, 33(6): 798-802.

[36] He SJ, Gu YH, Liao ZH. Relationship between GSTM1 gene polymorphism, smoking and drinking and susceptibility to primary liver cancer [J]. Journal of Guangxi Medical University, 2008, 25(4): 567-568.

[37] Yang D. Association between hedgehog signaling pathway gene promoter genetic variation and the susceptibility to hepatocellular carcinoma[D]. Guangxi: Guangxi Medical University, 2015.

[38] Qin RY. Association of hEXO1gene polymorphism with hepatocellular carcinoma and its clinical characteristics[D]. Guangxi: Guangxi Medical University, 2016.

[39] Chang Z. Association of HLA, GLB1 genetic polymorphisms with hepatocellular carcinoma susceptibility and prognosis of hepatocellular carcinoma patients [D]. Henan: Zhengzhou University, 2018.

[40] Han YN. Study of hMSH3 and hMSH2 gene single nucleotide polymorphism and environment interactions with susceptibility of hepatocellular carcinoma[D]. Hebei: North China University of Science and Technology, 2019.

[41] Wang AZ, Cong WM, He XL et al. A hOGG1 gene polymorphism and genetic susceptibility to colorectal cancer and hepatocellular carcinoma[J]. Chinese Journal of Gastroenterology and Hepatology, 2008, 17(10): 854-857.

[42] Gao M. Correlation between HSP90, SIRT3 gene polymorphism and the risk of liver cancer and between their expression and invasion in Hepatoma cells[D]. Anhui: Anhui Medical University, 2016.

[43] Zhu ZZ, Cong WM, Wang AZ, et al. Correlation between IGF2R polymorphism and genetic susceptibility to colorectal cancer and hepatocellular carcinoma [J]. Academic Journal of Naval Medical University, 2008, 29(11): 1289-1292.

[44] Wang XL. The study of genetic association between single nucleotide polymorphisms if IL-6, IL-10, KIF1B and susceptibility to primary hepatic carcinoma[D]. Guangdong: Guangdong Pharmaceutical University, 2013.

[45] Liu L, Liu Z, Xu Y, et al. Association between IL12 polymorphism and primary hepatocellular carcinoma in Chinese population [J]. Journal of Nanjing Medical University, 2010, 30(1): 34-38.

[46] Bei YC. The association between single nucleotide polymorphisms of IL-6, IL-10 and susceptibility to HBV-related hepatocellular carcinoma[D]. Guangxi: Guangxi Medical University, 2011.

[47] Hou B, Pei RF. Correlation between IL-8-251A/T polymorphism and liver cancer [J]. Chinese Journal of Gerontology, 2012, 32(13): 2736-2737.

[48] Lu XH, Zhu XQ, Zhang YY, et al. Correlation study on IL-8 Gene-251T/A, +781C/T polymorphisms and genetic susceptibility to hepatocellular carcinoma in Nantong area population [J]. Journal of Interventional Radiology, 2015, (4): 314-319.

[49] Tang WJ. Association between IL-6 genetic variation, Hepatic fibrosis and risk of liver diseases[D]. Guangxi: Guangxi Medical University, 2016.

[50] Zhang QX. Association of IL-21 and IL-18 gene polymorphisms with hepatocellular carcinoma caused by the hepatitis B virus[D]. Chongqing: Chongqing Medical University, 2016.

[51] Wang Y. Correlation between IL-28B gene polymorphisms and hepatocellular carcinoma[D]. Guangxi: Guilin Medical College, 2012.

[52] Chen Q, Liao WJ, He SQ, et al. The correlation between single nucleotide polymorphism omterleukin-18 gene promoter and genetic susceptibility to hepatocellular carcinoma[J]. Immunological Journal, 2012, 28(12): 1051-1055.

[53] Xie GX, Yin JH, Zhang Q, et al. Association of genetic polymorphisms of key molecules in JAK/STAT signaling pathway with susceptibility of hepatocellular carcinoma[J]. Chinese Journal of Epidemiology, 2012, (02): 215-219.

[54] Chen S. Association between 3'-UTR miRNA binding site polymorphisms in LIG4 gene and hepatocellular carcinoma[D]. Guangxi: Guangxi Medical University, 2019.

[55] Wang X, Zhang GL, Zhang YF, et al. Association between single nucleotide polymorphisms in MDM2 and the susceptibility of hepatocellular carcinoma [J]. Journal of Modern Oncology, 2013, 21(8): 1801-1804.

[56] Qiu MQ, Yu XY, Qin LY, et al. Association of Met gene polymorphisms with the risk of hepatocellular carcinoma [J]. Chinese Journal of Cancer Prevention and Treatment, 2015, 22(21): 1649-1653.

[57] Zhou Z, Yang DH. he association of the SNP in miRNA146a with genetic prediposion and the earlier recurrence after resection for hepatocellular carcinoma [J]. Chinese Journal of Hepatobiliary Surgery 2014, 20(5): 338-341.

[58] Cai M, Zheng WW, Zhang J, et al. Genetic polymorphisms of microRNA-4293 and hepatocellular carcinoma susceptibility in female [J]. Chinese Hepatology, 2016, 21(5): 347-350.

[59] Meng JF. Genetic association of microRNA biogenesis genes polymorphisms with the nasopharyngeal carcinoma and hepatitis B virus related hepatocellular carcinoma[D]. Anhui: Anhui Medical University, 2012.

[60] Li YC. MicroRNA related SNPs and genetic susceptibility to hepatocellular carcinoma[D]. Henan: Zhengzhou University, 2012.

[61] Wang Y, Duan X, Li K. Association of genetic variation in the promoter region of miR-22 gene and risk of HBV-related hepatocellular carcinoma[J]. China Journal of General Surgery, 2014, 23(1): 43-47.

[62] Pu R. The role of a polymorphism in promoter region of miR-34b/c and hepatitis B virus mutations in HBV-positive hepatocellular carcinoma[D]. [硕士学位论文]. Shanghai: Second Military Medical University, 2012.

[63] Wang WZ. Study on the association between miR-146a single nucleotide polymorphism and genetic susceptibility to liver cancer and microarray analysis of tumor-associated microRNA [D]. Shanghai: Fudan University, 2011.

[64] Duan L. Study of the correlation of miR-146a expression and rs2910164 polymorphism with liver cancer risk[D]. Hubei: Wuhan University, 2018.

[65] Bei CH. The association of the SNPs in miRNA-122 and miRNA-199 with hepatocellular carcinoma risk and prognosis[D]. Guangxi: Guangxi Medical University, 2015.

[66] Min P. Effect of MiRNA1269a single nucleotide polymorphism on susceptibility to liver cancer [D]. Fujian: Xiamen University, 2016.

[67] Tan SK, Qiu XQ, Tang GY, et al. Relationship between hepatocellular carcinoma and the interaction between NQO1 polymorphisms and environmental factors [J]. Chinese Journal of Hepatology, 2012, (11): 833-837.

[68] Wang WW. Association of NQO1 gene polymorphism with hepatocellular carcinoma[D]. Guangxi: Guilin Medical College, 2012.

[69] Zhu XY. Zhang W. Relationship between p21, p53 expression and single nucleotide polymorphism with hepatocellular carcinoma [J]. China Modern Doctor, 2013, 51(17): 6-9.

[70] Wang SQ. p53, MDM2 polymorphism are associated with the risk of hepatocellular carcinoma and therapeutic outcome with transcatheter arterial chemoembolization[D]. Liaoning: China Medical University, 2013.

[71] Mou NN, Zhang L. Effects of p53 gene codon 72 Arg/Pro polymorphisms on susceptibility of hepatocellular carcinoma in patients with hepatitis B virus infection in Shandong Province [J]. Journal of Hepatopancreatobiliary Surgery, 2013, 25(05): 381-384.

[72] Jiang DK. The relationship between mutation of p53 gene, R72P polymorphism and SNP309 polymorphism of MDM2 gene and the occurrence and prognosis of hepatocellular carcinoma [D]. Shanghai: Fudan University, 2008.

[73] Zhang XA. Genetic association between the polymorphisms of p53 pathway genes and susceptibility to hepatocellular carcinoma and nasopharyngeal carcinoma[D]. Beijing: Academy of Military Medical Sciences, 2008.

[74] Wang H, Wang H, Qiao LJ. Study on p73 gene polymorphism and genetic susceptibility to hepatocellular cancer [J]. Clinical Medicine of China, 2017, 33(3): 236-241.

[75] Zhang HY. The association and mechanism of patatin-like phospholipase domain-containing 3 gene and hepatitis B virus associated hepatocellular carcinoma[D]. Shandong: Qingdao University, 2016.

[76] Gao C, Wang X, Li LH, et al. The correlation study of PNPLA3 gene polymorphism and genetic susceptibility to hepatocellular carcinoma[J]. Medical Innovation of China, 2014, (18): 12-14.

[77] Tang YM. Association study on the polymorphisms of PPARγ gene with the susceptibility of hepatocellular carcinoma[D]. Guangxi: Guangxi Medical University, 2010.

[78] Wang JW. Association between genetic variation in miRNA target sites of RAD51D gene and susceptibility to hepatocellular carcinoma[D]. Guangxi: Guilin Medical College, 2018.

[79] Zhao JY. Study on associations between RANTES polymorphisms and HBV-related liver disease susceptibility[D]. Guangxi: Guangxi Medical University, 2015.

[80] Meng JF, Li PY, Zhang HX, et al. Association between RAN polymorphisms and susceptibility to hepatitis B related hepatocellular carcinoma in Guangxi [J]. Acta Universitatis Medicinalis Anhui, 2012, 47(09): 1070-1074.

[81] Chen QZ. Correlational analysis of the Regulome—SNP and primary hepatic cancer susceptibility[D]. Jilin: Yanbian University, 2019.

[82] Fan JJ, Chang CF, Wang H. Association of single-nucleotide polymorphisms of STAT3and STAT4 with hepatocellular carcinoma [J]. The Journal of Practical Medicine, 2018, 34(21): 3593-3597+3602.

[83] Fan JJ, Chen Y, Zhang W, et al. Effect of STAT3 gene polymorphism and its interaction with HBV precore mutation on the risk of hepatocellular carcinoma [J]. Chinese Journal of Integrated Traditional and Western Medicine on Digestion, 2019, 27(01): 40-45.

[84] Liu Y, Yu DC, Pan MJ, et al. A case-control study on the relationship between polymorphisms of STAT3 and XRCC4 gene and the risk of hepatocellular carcinoma[J]. International Journal of Laboratory Medicine, 2014, 35(07): 850-852.

[85] Pan MJ, Liu Y, Zhou YH. Relationship between polymorphisms of STAT4 and the susceptibility to hepatocellular carcinoma associated with hepatitis B virus [J]. The Practical Journal of Cance, 2016, 31(05): 704-706+733.

[86] Li L. Association between insertion/deletion polymorphisms in the 3’UTR of STN1 with hepatocellular carcinoma susceptibility in a Chinese population [D]. Jiangsu: Suzhou University, 2016

[87] Li YH. The association of surviving and COX-2 gene with hepatocellular carcinoma[D]. Shanghai: Fudan University, 2011.

[88] Li WY, Zhang Z, Li A, et al. Relationship between single nucleotide polymorphism of TLR4 gene rs7873784 and genetic susceptibility of liver cancer [J]. Journal of HeBei United University (Health Sciences), 2020, 22(5): 337-340+347.

[89] Liao YJ. The potential functional variants in TLR4 promoter region and gene-environment interactions contribute to genetic susceptibility of primary liver cancer: a case-control study in Shunde area[D]. Guangdong: Guangdong Pharmaceutical University, 2014.

[90] Lin XH. Genetic polymorphisms in TLR9 and gene-environment interactions contribute to susceptibility of primary liver cancer in Shunde population[D]. Guangdong: Guangdong Pharmaceutical University, 2015.

[91] Gao H, Wu HJ, Xie YN, et al. Association of TNFSF15 promoter -358 T>C genetic variation and risk of liver cancer[J]. The Chinese Journal of Clinical Pharmacology, 2019, 35(16): 1731-1734.

[92] Zhao JQ. Relationship between Toll-like receptor 2, 4, and 9 single nucleotide gene polymorphisms and susceptibility to hepatocellular carcinoma[D]. Shanghai: Shanghai Jiaotong University, 2011.

[93] Hao WL, Yang XY, Zhang AJ, et al. Association of TLR4 gene polymorphism with susceptibility to and prognosis of hepatitis B virus-related primary liver cancer [J]. Journal of Clinical Hepatology, 2020, 36(7): 1540-1544.

[94] Song W. Association between U2 dependent mRNA splicing complex related gene polymorphism and the risk of primary liver cancer[D]. Beijing: Peking Union Medical College, 2015.

[95] Liu Z. VDBP correlation between single nucleotide polymorphisms and the risk of hepatocellular carcinoma in China[D]. Liaoning: China Medical University, 2014.

[96] Hu HB. A case-control study of xpD gene polymorphism and external exposure factors and primary liver cancer in Guangxi Zhuang nationality [J]. China Health Care & Nutrition, 2017, 27(5): 56.

[97] Zeng XY, Yu HP, Qiu XQ, et al. A case-control study of polymorphism of XRCC1 gene and the risk of hepatocellular carcinoma [J]. Chinese Journal of Disease Control & Prevention, 2010: 760-763.

[98] Long XD. Polymorphisms at cording sequence of XRCC4 and XPC & hepatocellular carcinoma[D]. Shanghai: Shanghai Jiaotong University, 2012.

[99] Deng Y, Li RL, Guang XM, et al. A study of interleukin-16 gene polymorphism and genetic susceptibility to liver cancer[J]. The Journal of Practical Medicine, 2012, 28(23): 3910-3912.

[100] Wang HX. Epidermal growth factor gene polymorphisms associated with susceptibility to hepatocellular carcinoma[D]. Guangxi: Guangxi Medical University, 2009.

[101] Zhu GX. Analysis of on risk factors for hepatitis C virus related liber cirrhosis, hepatocellular carcinoma[D]. Shandong: Qingdao University, 2015.

[102] Meng PP, Wang F, Liu HM, et al. Analysis of Risk Factors affecting the progression from Hepatitis C Cirrhosis to Hepatocellular Carcinoma [J]. Chinese Journal of Integrated Traditional and Western Medicine on Liver Diseases, 2019, 29(04): 301-304.

[103] Tan C. Association of forkhead box O gene microRNA related SNPs with hepatocellular carcinoma and function validation of loci[D]. Guangxi: Guangxi Medical University, 2014.

[104] Zhou N, Zhang YR, Wei SF, et al. Association of ERα-29 gene polymorphisms with susceptibility to HBV-related hepatocellular carcinoma [J]. Journal of Clinical Hepatology, 2015, 31(2): 240-243.

[105] Zhang W. The correlation between gene polymorphisms of ER gene and primary liver cancer[D]. Chongqing: Chongqing Medical University, 2008.

[106] Zhou XJ. A case-control study on the association between polymorphisms of metabolic enzyme genes, external exposure factors and their interaction and hepatoma susceptibility[D]. Guangxi: Guangxi Medical University, 2011.

[107] Zhu ZZ. Studied on the relationship between single nucleotide polymorphisms and susceptibility to hepatocellular carcinoma in Chinese population[D]. Shanghai: Second Military Medical University, 2004.

[108] Lei L, Li X, He JH, et al. Interaction between MTHFR C677T polymorphism and alcohol consumption in hepatocarcinoma in Enshi area [J]. Hebei Medical Journal, 2016, 38(21): 3339-3340+3343.

[109] Yu HF, Gao YH, Ye XH. Analysis of risk factors for primary liver cancer in Leliu town of Shunde districe in Foshan [J]. Chinese Primary Health Care, 2014, 28(7): 95-98.

[110] Cai L, Yi YN. Epidemiological study on risk factors of primary liver cancer in Fujian Province[J]. Chinese Journal of Preventive Medicine, 1997, (9): 58-59.

[111] Luo HZ, Mi DH, Li YG, et al. Investigation of risk factors in the liver cancer of Wuwei city [J]. Cancer Research and Clinic, 2010, 22(2): 134-137.

[112] Huang TJ. Epidemiologic study on the risk factor of hepatoma[D]. Fujian: Fujian Medical University, 2009.

[113] Xu J. Genetic variations in microRNA-binding site of genes dysregulated in hepatocellular carcinoma and serum microRNAs in patients with hepatocellular carcinoma[D]. Beijing: Peking Union Medical College, 2008.

[114] Zhang YC, Chen YP, Chen JX, et al. Study on the polymorphisms and promoter methylation and expression of the glutathione S-transferases P1 gene in hepatocellular carcinoma [J]. Chinese Journal of Digestion, 2006, 26(7): 468-472.

[115] Zhang ZH, Jiang FX, He Y. Risk factors analysis of liver cancer caused by cirrhosis after hepatitis [J]. Hainan Medical Journal, 2014, 25(1): 24-26.

[116] Tang GT, Li XP, Liu TQ et al. A study of genetic polymorphisms of Glutathione S-transferase in patients with hepatocellular carcinoma [J]. Chinese Journal of Laboratory Diagnosis, 2012, 16(04): 660-662.

[117] Lin ZB, Qi YF, Zhou XF, et al. Analysis of the risk factors for primary liver cancer in Shunde region, Guangdong [J]. Chinese Journal of Disease Control & Prevention, 2017, 21(10): 993-996+1001.

[118] Ye R, Shao YH, Wu ZQ, et al. Study on the risk factors and familial correlation of primary hepatocellular carcinoma (PHC) in Shunde, Guangdong[J]. Journal of Guangdong Pharmaceutical University, 2009, 25(6): 628-631.

[119] Long XD, Ma Y, Wei YP, et al. Study on the detoxication gene gstM1-, gstT1-null and susceptibility to aflatoxin B1-related hepatocellular carcinoma in Guangxi [J]. Chinese Journal of Epidemiology, 2005, (10): 53-57.

[120] Yang Y. The genetic polymorphism of cytokine genes in Guangxi and hepatocellular carcinoma susceptibility[D]. Guangxi: Guangxi Medical University, 2011.

[121] Wu H. Study on single nucleotide polymorphisms of XRCC1 and Caspase9 genes and genetic susceptibility to hepatocellular carcinoma in Fusui population of Guangxi Province[D]. Guangxi: Guangxi Medical University, 2009.

[122] Ji L. A case control study on risk factors of hepatocellular carcinoma in Guangxi[J]. Journal of Cancer Control and Treatment, 2010, 23(06): 453-457.

[123] Tan SK, Qiu XQ, Yu HP, et al. Etiologic fraction and interaction of risk factors for primary hepatic carcinoma in Guangxi, China [J]. Chinese Journal of Preventive Medicine, 2008, 42(3): 169-172.

[124] Lan YJ. Primary liver cancer risk factors analysis Guangzhou in Guangxi region[D]. Guangxi: Guangxi Medical University, 2015.

[125] Wu XM. DNA repair gene XPC Lys939Gln polymorphism & susceptibility to aflatoxin B1-related hepatocellular carcinoma risk in Guangxi population[D]. Guangxi: Guangxi Medical University, 2010.

[126] Huang QH, Huang TR, Li JL, et al. Correlation between microRNA-146a polymorphism and primary liver carcinoma in the Guangxi Zhuang population [J]. Chinese Journal of Oncology Prevention and Treatment, 2013, (2): 100-104.

[127] Zhao XK, Cheng ML, Zhang Q, et al. A case-control study on the risk factors of hepatocellular carcinoma in Guizhou Province [J]. Chinese Journal of Hepatology, 2014, 22(1): 33-37.

[128] Xu DF, Zhou KL, Li ZR, et al. Analysis of miRNA146a single nucleotide polymorphism and susceptibility of patients with hepatocellular carcinoma of Li nationality in Hainan Province[J]. Chinese Journal of Liver Diseases (Electronic), 2019, 11(02): 21-24.

[129] Zhang JY, Han SG, Dai M, et al. A case-control study of risk factors for liver cancer in Henan province[J]. Chinese Journal of Public Health, 1997, (9): 338-340.

[130] Pei GJ. Study on risk factors of hepatocellular carcinoma in Kaifeng County, Henan province[D]. Henan: Zhengzhou University, 2008.

[131] Liu LF, Zhang JL, Lin JS. The relationship between Cyciooxygenase-2 gene-1195G/A genotype and risk of HBV-induced HCC: a case-control study in Han Chinese people [J]. Chinese Journal of Gastroenterology and Hepatology, 2010, 19(4): 333-335.

[132] Chen XP. Single nucleotide polymorphisms in aflatoxin B1 response related genes: implication in evolution genetics, relevance in pharmacogenetics, and association with hepatocellular carcinoma[D]. Beijing: PLA Academy of Military Medical Sciences, 2005.

[133] Lin ZF. The association between genetic variation of lncRNA based on GWAS and outcome of HBV infection[D]. Guangdong: Guangdong Pharmaceutical University, 2017.

[134] Zhang ST. Candidate SNPs-based study on susceptibility to hepatitis C virus-related liver cirrhosis and hepatocellular carcinoma[D]. Tianjin: Tianjin Medical University, 2017.

[135] Yuan XY. Genetic associated study between candidate genes polymorphisms and hepatocellular carcinoma in Chinese population and functional study of SNPs in the promoter of CD8[D]. Chongqing: Third Military Medical University, 2007.

[136] Hong YB, Zhang T, Sun LG. Association between IL-2+114(G/T) polymorphism and susceptibility to hepatitis B, cirrhosis, and hepatocellular carcinoma in Jilin province[J]. Journal of Clinical Hepatology, 2012, 28(12): 930-933.

[137] Miao XY, Xu L, Liu H, et al. Association of MICA polymorphism with HBV-related hepatocellular carcinoma among Han population in Jiangsu[J]. Laboratory Medicine and Clinic, 2014, (18): 2548-2549+2552.

[138] Cao YY. Study on genetic and environmental factors in primary liver cancer in Luoyang, China[D]. Shanghai: Fudan University, 2004.

[139] Wang L, Wang P, Li XD. Investigation on prevalence of liver cancer and its influencing factors in patients with chronic hepatitis[J]. Journal of Clinical Psychosomatic Diseases, 2019, 25(1): 51-54.

[140] Zhou LX, Han T. Risk factors of hepatocellular carcinoma in patients with hepatitis B virus-related liver cirrhosis[J]. Journal of Tianjin Medical University, 2017, 23(03): 214-216.

[141] Zhang YQ, Peng LJ, Cao YR, et al. Analysis of the risk factors of hepatocellular carcinoma in cirrhotic patients with chronic hepatitis B[J]. Chinese Journal of Hepatology, 2015, 23(07): 512-516.

[142] Chen QF, Luo XM, Zhao MP, et al. Analysis on influencing factors of chronic hepatitis B patients complicated with primary hepatic carcinoma [J]. Chinese Journal of Public Health Management, 2018, 34(1): 79-82.

[143] Xu HX, Li XH. Susceptibility of Microrna-149 and 196A2 Single Nucleotide Polymorphisms to HCC in Mongolian [J]. Journal of Kunming Medical University, 2020, 41(06): 132-135.

[144] Lin QB. The risk factors of HBV related hepatocellular carcinoma in the men aged≤40 year[D]. Fujian: Fujian Medical University, 2013.

[145] Wang FF, Cui LH, Song Y, et al. Related factors of hepatocellular carcinoma among Qingdao citizens: a case-control study [J]. Chinese Journal of Public Health, 2011, 27(11): 1420-1422.

[146] Pan HH, Su CH, Lin Y, et al. Association between rs17401966 polymorphism of 1B gene of driver protein family member and genetic susceptibility to primary hepatocellular carcinoma [J]. Chinese Journal of Preventive Medicine, 2015, 49(05): 419-423.

[147] Yu WB. Association study between polymorphism at 6q25.1 and susceptibility and prognosis of hepatocellular carcinoma[D]. Beijing: Peking Union Medical College, 2014.

[148] Wang XY, Sun YJ, Zhang TT, et al. Association between Human leukocyte antigen DRA gene polymorphisms and susceptibility to hepatocellular carcinoma [J]. Modern Preventive Medicine, 2020, 47(23): 4404-4407.

[149] Su CH. The association study between HBV infection, environmental factors, polymorphisms and the risk of hepatocellular carcinoma in Xiamen[D]. Fujian: Fujian Medical University, 2015.

[150] Pan HH. Association between P53 gene and MDM2 gene polymorphism and genetic susceptibility to primary hepatocellular carcinoma in Xiamen City[D]. Fujian: Xiamen University, 2015.

[151] Zhou XF, Wei ZM, Zhou CY, et al. Analysis of influencing factors for liver cancer by gender in Shunde region[J]. Chinese Journal of Disease Control & Prevention, 2019, 23(2): 129-133+139.

[152] Zhang XW, Pan SD, Feng YL, et al. Relationship between genetic polymorphism in microRNAs precursor and genetic prediposition of hepatocellular carcinoma [J]. Chinese Journal of Preventive Medicine, 2011, 45(3): 239-243.

[153] Zou CL, Chen ZJ, Jin WY, et al. Etiologic fraction and interaction of risk factors for primary hepatocellular carcinoma in Wenzhou, Zhejiang Province[J]. Chinese Journal of Preventive Medicine, 2003, 37(5): 355-357.

[154] Xin SJ, Zheng LS, Wang DD, et al. Analysis on association between single nucleotide polymorphism of selenoprotein S gene and risk of liver cancer[J]. Journal of Jilin University Medicine Edition), 2018, 44(3): 558-562.

[155] Zhao B, Guo LB, Duan WQ. Association of cytotoxic T lymphocyte antigen 4 gene polymorphisms with liver cancer susceptibility [J]. Chinese Hepatology, 2019, 24(12): 1453-1455.

[156] Cui WL, Li N, Yu J, et al. Association of CTLA4 polymorphysims with susceptibility to hepatocellular carcinoma[J]. China Journal of Modern Medicine, 2016, (2): 24-28.

[157] Wu XH, Yang XY, Duan W, et al. The relationship between genetic polymorphism of cytochrome p450 and susceptibility to hepatocellular carcinoma[J]. Journal of Logistics University of PAP (Medical Sciences) 2007, 16(1): 47-48+62.

[158] Xu C. Association between genetic polymorphisms of cytochrome P450 oxidoreductase and hepatocellular carcinoma susceptibility[D]. Henan: Zhengzhou University, 2019.

[159] Zhu ZZ, Cong WM, Xian ZH, et al. Correlation of Cyclin D1 polymorphism with genetic susceptibility to hepatocellular carcinoma[J]. Chinese Journal of Cancer Prevention and Treatment, 2007, (20): 1521-1523.

[160] Liu S. Single nucleotide polymorphism in sex hormone-binding globulin and IL=6 genes and the incidence of hepatocellular carcinoma[D]. Guangxi: Guangxi Medical University, 2012.

[161] Xiao Q, Wu XF, Ye H, et al. Association between IL-8-251T＞A polymorphisms and hepatocellular carcinoma[J]. Anatomy Research, 2014, 36(04): 274-276.

[162] Li HB. Correlation between AKT2 gene polymorphism with primary hepatocellular carcinoma in Yanbian area[D]. Jilin: Yanbian University, 2017.

[163] Liu CZ. Study on relationship between genetic polymorphism of drug metabolizing enzymes and susceptibility to primary hepatocellular carcinoma[D]. Shanghai: Fudan University, 2001.

[164] Xu MJ, Gu ZY, Zhao JY et al. Association study on genetic polymorphisms of folate metabolism genes and susceptibility of hepatocellular carcinoma[J]. Journal of Fudan University (Natural Science), 2014, 53(6): 716-723.

[165] An Y. Association study on polymorphisms of one-carbon pathway with susceptibility of hepatocellular carcinoma[D]. Shanghai: Fudan University, 2008.

[166] Cai M. The genetic association between IGF pathway genes and HBV-related HCC[D]. Beijing: Beijing University of Technology, 2012.

[167] Yan H, Yan N. Analysis of risk factors of hepatitis B cirrhosis complicated with primary liver cancer [J]. Journal of Qiqihar University of Medicine, 2015, (19): 2911-2912.

[168] Zhan CL. Logistic regression analysis of risk factors of hepatitis cirrhosis complicated with hepatocellular carcinoma[J]. Journal of Clinical Research, 2016, 33(9): 1697-1701.

[169] Chen P. Analysis of risk factors associated with liver cirrhosis of hepatocellular carcinoma[D]. Anhui: Anhui Medical University, 2013.

[170] Wang XL. Analysis of related risk factors of hepatitis B-induced liver cirrhosis complicated with primary hepatic carcinoma[J]. China Practical Medical, 2015, (16): 38-40.

[171] Guo JX, Wei XH. Risk factors and prognosis of hepatitis B cirrhosis patients with primary liver cancer[J]. Shaanxi Medical Journal, 2019, 48(7): 850-852.

[172] Lin LY. Investigation of primary liver carcinoma in patients with hepatitis B and its influencing factors[J]. Journal of Public Health and Preventive Medicine, 2020, 31(5): 114-117.

[173] Sun GZ. Risk and prevention of liver cancer in hepatitis B patients[J]. The Journal of Medical Theory and Practice, 2015, (2): 203-204.

[174] Hong WM. Analysis of influencing factors of viral hepatitis B complicated with primary liver cancer[J]. Chinese Journal of Rural Medicine and Pharmacy, 2019, 26(24): 18-19.

[175] Guo TZ, Han LX, Luo Y, et al. Risk factors of liver cancer in patients with hepatitis B virus infection[J]. International Journal of Virology, 2016, 23(1): 26-28.

[176] Wang YQ, Chen CJ, Zhang GW. Analysis of risk factors of hepatitis B virus-associated primary liver cancer[J]. Chinese Hepatology, 2019, 24(10): 1180-1182.

[177] Zhang R, Zhu LY, Zhao Y. Risk factors of hepatitis B cirrhosis complicated with liver cancer[J]. Infectious Disease Information, 2019, 32(6): 539-541.

[178] Shi CC, Xue F, Sun YF, et al. Evaluation of Risk Factors of Primary Hepatic Carcinoma Due to Hepatitis B Cirrhosis [J]. The Practical Journal of Cancer, 2013, 28(4): 399-401.

[179] Li L, Yang WW, Wei T, et al. Logistic analysis of risk factors of hepatitis B cirrhosis complicated with primary liver cancer[J]. Chinese Journal of Gerontology, 2016, 36(7): 1653-1654.

[180] Yang XM, Zhang W, Zheng HB, et al. Analysis of risk factors for hepatitis B liver cirrhosis complicated with primary liver cancer[J]. China Modern Doctor, 2013, 51(7): 54-55+58.

[181] Chen P, Li J, Su F, et al. Risk factors for hepatocellular carcinoma in patients with liver cirrhosis[J]. Acta Universitatis Medicinalis Anhui, 2012, 47(10): 1218-1221.

[182] Wu CX, Zhou XL, Chen YH, et al. Risk factors for occurrence of hepatocellular carcinoma in patients with chronic hepatitis B[J]. Journal of Practical Hepatology, 2015, 18(3): 306-307.

[183] Gao R, Liu ZJ, Chen F, et al. Multivariate regression analysis of risk factors for HBV-related primary liver cancer[J]. Journal of Clinical Hepatology, 2014, (4): 370-372.

[184] Sun XY, Wang BY. The effect of age, gender and alcohol consumption to pathogecy of hepatic carcinoma[J]. Drug Evaluation, 2009, 6(5): 197-199+189.

[185] Li H, Liu SY, Liu J, et al. Study on relationship between alcohol drinking, HBsAg and HCC[J]. Journal of Medical Forum, 2015, 36(5): 12-14.

[186] Yu SZ, Mu LN, Cai L, et al. The drinking water and three environmental risk factors for hepatocellular carcinoma in Taixing by case-control study[J]. Fudan University Journal of Medical Sciences, 2008, 35(1): 31-38.

[187] Dong SB, Wang FZ, Zhang S, et al. Sequence analysis of HBV in primary hepatomas patients infected with HBV[J]. Chinese Journal of Experimental and Clinical Virology, 2017, 31(2): 92-97.

[188] Tang GT, Li XP, Liu TQ, et al. Genetic polymorphisms of DNA repair genes in patients with hepatocellular carcinoma[J]. Shandong Medical Journal, 2011, 51(42): 19-20.

[189] Tang GT, Li XP, Liu TQ, et al. Polymorphism of nuclear factor κB signal transduction pathway gene in primary hepatocellular carcinoma population[J]. Guangdong Medical Journal, 2012, 33(8): 1126-1128.

[190] Kuang JX, Gan HW, Li RJ, et al. Relationship between primary hepatocellular carcinoma and HBV, HCV infection[J]. China Medical Herald, 2010, 07(33): 42-43.

[191] He J. Study on the correlation between primary liver cancer and abnormal glucose metabolism [D]. Guangdong: Shantou University, 2012.

[192] Bo H, Qiu XQ, Liu S, et al. Gene polymorphism of IL-2, IFN-gamma and primary hepatocellular carcinoma[J]. Chinese Journal of Public Health, 2012, 28(09): 1140-1144.

[193] Fan JJ, Chang CF, Wang H. Correlation between single-nucleotide polymorphisms of interleukin-28B and hepatocellular carcinoma, Zhangjiakou[J]. Modern Preventive Medicine, 2018, 45(19): 3629-3633+3638.

[194] Wang WW, Chen GY, Sun JJ, et al. The relationship between polymorphisms of NQO1 genes and hepatocellular carcinoma in Zhengzhou and Guilin areas [J]. Chinese Journal of Hepatobiliary Surgery, 2013, 19(11): 836-840.

[195] Guo WY, Dai L, Pu JY, et al. Association of genetic variant in 12-lipoxygenase and risk of hepatic carcinoma[J]. National Medical Journal of China, 2013, 93(46): 3685-3687.

[196] Ning BS, Bing H, Wang W, et al. The association of PNPLA3 rs738409 SNP with primary hepatocellular carcinoma in Chinese Han population[J]. Progress of Anatomical Sciences, 2019, 25(2): 157-160+165.

[197] Li ML, Li YC, Zhao HH, et al. The relationship between the hepatic lipase gene 250G/A polymorphism and the risk of hepatocellular carcinoma in Chinese population [J]. Journal of Clinical and Pathological Research, 2016, 36(09): 1399-1405.

[198] Zhang HX. Genetic association of genetic polymorphisms with SARS and hepatocellular carcinoma in Chinese population[D]. Beijing: Institute of Biophysics, Chinese Academy of Sciences, 2008.

[199] Huang GY, Ma XG, Wang CC. Heavy alcohol consumption can enhance risk of hepatocellular carcinoma associated with hepatitis B virus infection [J]. Chinese Journal of Cancer Prevention and Treatment, 2005, 12(6): 405-408.

[200] Wei ZH, Lu JX, Pu J, et al. Association of the TGF-β1 gene promoter polymorphisms with hepatocellular carcinoma [J]. Cancer Research and Clinic, 2012, 24(7): 447-450.

[201] Wu X, Wu J, Xin Z, et al. A 3′ UTR SNP in COL18A1 is associated with susceptibility to HBV related hepatocellular carcinoma in chinese: Three independent case-control studies[J]. PLoS One, 2012, 7(3): e33855.

[202] Dong D, Gao X, Zhu Z, et al. A 40-bp insertion/deletion polymorphism in the constitutive promoter of MDM2 confers risk for hepatocellular carcinoma in a Chinese population[J]. Gene, 2012, 497(1): 66-70.

[203] Niu CZ, Zhang FH, Li Y, et al. The -250G/A and -514C/T polymorphisms in hepatic lipase gene promoter confers an increased risk of hepatocellular carcinoma in a Chinese population[J]. Ann Hepatol, 2018, 17(6): 992-1000.

[204] Qi P, Chen YM, Wang H, et al. -509C>T polymorphism in the TGF-β1 gene promoter, impact on the hepatocellular carcinoma risk in Chinese patients with chronic hepatitis B virus infection[J]. Cancer Immunol Immunother, 2009, 58(9): 1433-1440.

[205] Li Q, Yu CH, Yu JH, et al. ABO blood group and the risk of hepatocellular carcinoma: A case-control study in patients with chronic hepatitis B[J]. PLoS One, 2012, 7(1): e5587.

[206] Chen PY, Fang AP, Wang XY, et al. Adherence to the Chinese or American Dietary Guidelines is Associated with a Lower Risk of Primary Liver Cancer in China: A Case-Control Study[J]. Nutrients, 2018, 10(8):

[207] Liu J, Yang H-I, Lee MH, et al. Alcohol Drinking Mediates the Association between Polymorphisms of ADH1B and ALDH2 and Hepatitis B-Related Hepatocellular Carcinoma[J]. Cancer Epidemiol Biomarkers Prev, 2016, 25(4): 693-699.

[208] Koh WP, Yuan JM, Wang R, et al. Aromatase (CYP19) Promoter Gene Polymorphism and Risk of Non-Viral Hepatitis-Related Hepatocellular Carcinoma[J]. Cancer, 2011, 117(15): 3383-3392.

[209] Yao X, Zeng H, Zhang G, et al. The associated ion between the VDR gene polymorphisms and susceptibility to hepatocellular carcinoma and the clinicopathological features in subjects infected with HBV[J]. BioMed Res Int, 2013, 2013: 953974.

[210] Yang D, Zhou F, Wang X, et al. Association analysis between MDR1 gene polymorphisms and risk of hepatocellular carcinoma in Chinese population[J]. Biomarkers, 2013, 18(3): 236-241.

[211] Liu Y, Zhang A, Liu Y, et al. Association analysis between the c.1804C>A genetic polymorphism of XRCC1 gene and risk of hepatocellular carcinoma in Chinese population[J]. Med Oncol, 2014, 31(3): 854.

[212] Li XF, Chen YX, Ye WW, et al. Association between a single nucleotide polymorphism of the XRCC1 gene and hepatocellular carcinoma susceptibility in the Chinese Han population[J]. Genet Mol Res, 2014, 13(1): 160-166.

[213] Li C, Xiong Y, Zhong Z, et al. Association Between a Variant in ADAMTS5 and the Susceptibility to Hepatocellular Carcinoma in a Chinese Han Population[J]. Cell Biochem Biophys, 2015, 72(1): 221-225.

[214] Wang R, Zhang J, Jiang W, et al. Association between a variant in MicroRNA-646 and the susceptibility to hepatocellular carcinoma in a large-scale population[J]. Sci World J, 2014, 2014: 312704.

[215] Li X, Xu H, Ding Z, et al. Association between ABO blood group and HCV-related hepatocellular carcinoma risk in China[J]. Medicine, 2016, 95(49): e5587.

[216] Wang C, Zhao H, Zhao X, et al. Association between an insertion/deletion polymorphism within 3′UTR of SGSM3 and risk of hepatocellular carcinoma[J]. Tumour Biol, 2014, 35(1): 295-301.

[217] He X, Xu H, Wang X, et al. Association between APOBEC3B deletion polymorphism and susceptibility to chronic hepatitis B infection and outcomes of hepatocellular carcinoma in Chinese Han population[J]. Int J Clin Exp Pathol, 2016, 9(9): 9520-9528.

[218] Liu Y, Xie L, Zhao J, et al. Association between catalase gene polymorphisms and risk of chronic hepatitis B, hepatitis B virus-related liver cirrhosis and hepatocellular carcinoma in Guangxi population: a case-control study[J]. Medicine (Baltimore), 2015, 94(13): e702.

[219] Liu Z, Song Z, Sun J, et al. Association between CTLA-4 rs231775 polymorphism and hepatocellular carcinoma susceptibility[J]. Int J Clin Exp Pathol, 2015, 8(11): 15118-15122.

[220] Li Y, Xie Q, Lu F, et al. Association between epidermal growth factor 61A/G polymorphism and hepatocellular carcinoma susceptibility in Chinese patients[J]. Liver Int, 2010, 30(1): 112-118.

[221] Yang Y, Zhou Y, Lu M, et al. Association between fibroblast growth factor receptor 4 polymorphisms and risk of hepatocellular carcinoma[J]. Mol Carcinog, 2012, 51(7): 515-521.

[222] Kou JT, Fan H, Han D, et al. Association between four common microRNA polymorphisms and the risk of hepatocellular carcinoma and HBV infection[J]. Oncol Lett, 2014, 8(3): 1255-1260.

[223] Bao J, Lu Y, Deng Y, et al. Association between IL-18 polymorphisms, serum levels, and HBV-related hepatocellular carcinoma in a Chinese population: A retrospective case-control study[J]. Cancer Cell Int, 2015, 15(1): 72.

[224] Tao R, Hu S, Wang S, et al. Association between indel polymorphism in the promoter region of lncRNA GAS5 and the risk of hepatocellular carcinoma[J]. Carcinogenesis, 2015, 36(10): 1136-1143.

[225] Lau HK, Hsieh MJ, Yang SF, et al. Association between interleukin-18 polymorphisms and hepatocellular carcinoma occurrence and clinical progression[J]. Int J Med Sci, 2016, 13(7): 556-561.

[226] Leu JD, Lin IF, Sun YF, et al. Association between MDM2-SNP309 and hepatocellular carcinoma in Taiwanese population[J]. World J Gastroenterol, 2009, 15(44): 5592-5597.

[227] Zhang S, Lin J, Jiang J, et al. Association between methylenetetrahydrofolate reductase tagging polymorphisms and susceptibility of hepatocellular carcinoma: A case–control study[J]. Biosci Rep, 2019, 39(11): BSR20192517.

[228] Li W, Ma Y, Zeng D, et al. Association between microRNA single nucleotide polymorphisms and the risk of hepatocellular carcinoma[J]. Rev Med Chil, 2016, 144(4): 508-515.

[229] Zhou B, Dong LP, Jing XY, et al. Association between miR-146aG>C and miR-196a2C>T polymorphisms and the risk of hepatocellular carcinoma in a Chinese population[J]. Tumour Biol, 2014, 35(8): 7775-7780.

[230] Wang Q, Yu X, Li Q, et al. Association between miR-199a rs74723057 and MET rs1621 polymorphisms and the risk of hepatocellular carcinoma[J]. Oncotarget, 2016, 7(48): 79365-79371.

[231] Duan S, Yu J, Han Z, et al. Association between P2RX7 gene and hepatocellular carcinoma susceptibility: A case-control study in a Chinese Han population[J]. Med Sci Monit, 2016, 22: 1916-1923.

[232] Gao X, Liu W, Yang L, et al. Association between PNPLA3 gene polymorphisms and risk of hepatitis B virus-related hepatocellular carcinoma in Han population in China:a case-control study[J]. Scand J Gastroenterol, 2017, 52(10): 1120-1127.

[233] Bei C, Tan C, Zhu X, et al. Association between Polymorphisms in CMTM Family Genes and Hepatocellular Carcinoma in Guangxi of China[J]. DNA Cell Biol, 2018, 37(8): 691-696.

[234] Chen LL, Shen Y, Zhang JB, et al. Association between polymorphisms in the promoter region of pri-miR-34b/c and risk of hepatocellular carcinoma[J]. Genet Mol Res, 2016, 15(4): gmr.15048723.

[235] Su C, Lin Y, Niu J, et al. Association between polymorphisms in tumor suppressor genes and oncogenes and risk of hepatocellular carcinoma: a case–control study in an HCC epidemic area within the Han Chinese population[J]. Med Oncol, 2014, 31(12): 1-6.

[236] Li J, Yang Q, He Z, et al. The association between polymorphisms of class ii cytokine receptor genes and risk of HBV-related hepatocellular carcinoma[J]. Int J Clin Exp Med, 2017, 10(8): 12492-12500.

[237] Shao YJ, Chan TS, Tsai K, et al. Association between proton pump inhibitors and the risk of hepatocellular carcinoma[J]. Aliment Pharmacol Ther, 2018, 48(4): 460-468.

[238] Zhang J, Wang R, Ma YY, et al. Association between single nucleotide polymorphisms in miRNA196a-2 and miRNA146a and susceptibility to hepatocellular carcinoma in a Chinese population[J]. Asian Pac J Cancer Prev, 2013, 14(11): 6427-6431.

[239] Qiu M, Liu Y, Zhou Z, et al. Association between Single-Nucleotide Polymorphism in MicroRNA Target Site of DDB2 and Risk of Hepatocellular Carcinoma in a Southern Chinese Population[J]. BioMed Res Int, 2020, 2020: 8528747.

[240] Deng X, Liang J, Jiang M, et al. Association between the c.1161G>A and c.1779C>G genetic variants of XRCC1 gene and hepatocellular carcinoma risk in Chinese population[J]. Int J Biol Sci, 2013, 9(3): 289-294.

[241] Wan YY, Wang XW, Hui HX, et al. Association between the c.1564A>T genetic polymorphism of the MDR1 gene and hepatocellular carcinoma in Chinese population[J]. Genet Mol Res, 2014, 13(3): 6820-6826.

[242] Li XF, He HB, Zhu YS, et al. Association between the c.3751G>A genetic variant of MDR1 and hepatocellular carcinoma risk in a Chinese Han population[J]. Asian Pac J Cancer Prev, 2013, 14(9): 5361-5365.

[243] Ma L, Chen S, Mao X, et al. The association between TNFR gene polymorphisms and the risk of Hepatitis B Virus-Related Liver Diseases in Chinese population[J]. Sci Rep, 2018, 8(1): 9240.

[244] Zhou J, Lv R, Song X, et al. Association between two genetic variants in miRNA and primary liver cancer risk in the Chinese population[J]. DNA Cell Biol, 2012, 31(4): 524-530.

[245] Li Z, Guo Y, Zhou L, et al. Association of a functional RAD52 genetic variant locating in a miRNA binding site with risk of HBV-related hepatocellular carcinoma[J]. Mol Carcinog, 2015, 54(9): 853-858.

[246] Deng B, Liu F, Wei Y, et al. Association of a p73 exon 2 G4C14-to-A4T14 polymorphism with risk of hepatocellular carcinoma in a Chinese population[J]. Tumour Biol, 2013, 34(1): 293-299.

[247] Qi P, Dou TH, Geng L, et al. Association of a variant in MIR 196A2 with susceptibility to hepatocellular carcinoma in male Chinese patients with chronic hepatitis B virus infection[J]. Hum Immunol, 2010, 71(6): 621-626.

[248] Zhang Y, Wang S, Wen X, et al. Association of ACYP2 and MPHOSPH6 genetic polymorphisms with the risk of hepatocellular carcinoma in chronic hepatitis B virus carriers[J]. Oncotarget, 2017, 8(49): 86011-86019.

[249] Deng Y, Chen ZJ, Lan F, et al. Association of CD44 polymorphisms and susceptibility to HBV-related hepatocellular carcinoma in the Chinese population[J]. J Clin Lab Anal, 2019, 33(8): e22977.

[250] Yang J, Liu J, Chen Y, et al. Association of CTLA-4 tagging polymorphisms and haplotypes with hepatocellular carcinoma risk: A case-control study[J]. Medicine, 2019, 98(29): e16266.

[251] Wang J-j, Wang Z-b, Tan T-c. Association of CTLA-4, TNF alpha and IL 10 polymorphisms with susceptibility to hepatocellular carcinoma[J]. Scand J Immunol, 2019, 90(6): e12819.

[252] Chang WS, Yang MD, Tsai CW, et al. Association of Cyclooxygenase 2 Single-Nucleotide Polymorphisms and Hepatocellular Carcinoma in Taiwan[J]. Chin J Physiol, 2012, 55(1): 1-7.

[253] Chen X, Wang H, Xie W, et al. Association of CYP1A2 genetic polymorphisms with hepatocellular carcinoma susceptibility: A case-control study in a high-risk region of China[J]. Pharmacogenet Genomics, 2006, 16(3): 219-227.

[254] Wu J, Zhang W, Xu A, et al. Association of epidermal growth factor and epidermal growth factor receptor polymorphisms with the risk of hepatitis b virus-related hepatocellular carcinoma in the population of north China[J]. Gent Test and Mol Biomarkers, 2013, 17(8): 595-600.

[255] Li X, Li K, Wu Z. Association of four common SNPs in microRNA polymorphisms with the risk of hepatocellular carcinoma[J]. Int J Clin Exp Pathol, 2015, 8(8): 9560-9566.

[256] Li H, Tang XM, Liu Y, et al. Association of Functional Genetic Variants of HOTAIR with Hepatocellular Carcinoma (HCC) Susceptibility in a Chinese Population[J]. Cell Physiol Biochem, 2017, 44(2): 447-454.

[257] Yue X, Jiang X, Zou H, et al. Association of hepatocellular carcinoma risk with polymorphisms in tumour necrosis factor alpha gene in a Chinese Han population[J]. Int J Immunogenet, 2020, 47(3): 286-293.

[258] Jiang Y, Chen S, Jia S, et al. Association of HLA-G 3′ UTR 14-bp insertion/deletion polymorphism with hepatocellular carcinoma susceptibility in a Chinese population[J]. DNA Cell Biol, 2011, 30(12): 1027-1032.

[259] Huang L, Liu C, Deng Y, et al. Association of hypoxia-inducible factor-2 alpha gene polymorphisms with the risk of hepatitis B virus-related liver disease in Guangxi Chinese: A case-control study[J]. PLoS One, 2016, 11(7): e0158241.

[260] Peng Q, Qin X, He Y, et al. Association of IL27 gene polymorphisms and HBV-related hepatocellular carcinoma risk in a Chinese population[J]. Infec Genet Evol, 2013, 16: 1-4.

[261] Wang JL, Nong LG, Wei YS, et al. Association of interleukin-8 gene polymorphisms with the risk of hepatocellular carcinoma[J]. Mol Biol Rep, 2014, 41(3): 1483-1489.

[262] Ren YQ, Han JQ, Cao JB, et al. Association of MDR1 gene polymorphisms with susceptibility to Hepatocellular carcinoma in the chinese population[J]. Asian Pac J Cancer Prev, 2012, 13(11): 5451-5454.

[263] Gao J. Association of MDR1 gene polymorphisms with the risk of hepatocellular carcinoma in the Chinese Han population[J]. Braz J Med Biol Res, 2013, 46(3): 311-317.

[264] Su SC, Ho YC, Liu YF, et al. Association of melatonin membrane receptor 1A/1B gene polymorphisms with the occurrence and metastasis of hepatocellular carcinoma[J]. Oncotarget, 2017, 8(49): 85655-85669.

[265] Zhang J, Wang R, Cai M, et al. Association of microRNA-3144 variant with the susceptibility to hepatocellular carcinoma[J]. Genes Genomics, 2014, 36(6): 771-776.

[266] Wang XH, Wang FR, Tang YF, et al. Association of miR-149C>T and miR-499A>G polymorphisms with the risk of hepatocellular carcinoma in the Chinese population[J]. Genet Mol Res, 2014, 13(3): 5048-5054.

[267] Zhao S, Yang Y, Liu J, et al. Association of mitochondrial DNA content in peripheral blood leukocyte with hepatitis B virus-related hepatocellular carcinoma in a Chinese Han population[J]. Cancer Science, 2011, 102(8): 1553-1558.

[268] Cai J, Cai Y, Ma Q, et al. Association of p53 codon 72 polymorphism with susceptibility to hepatocellular carcinoma in a Chinese population from northeast Sichuan[J]. Biomedical Reports, 2017, 6(2): 217-222.

[269] Lao X, Wang X, Liu Y, et al. Association of Paraoxonase 1 Gene Polymorphisms With the Risk of Hepatitis B Virus-related Liver Diseases in a Guangxi Population: A Case-control Study[J]. Medicine (Baltimore), 2015, 94(48): e2179.

[270] Ding J, Gao Y, Liu R, et al. Association of PTEN polymorphisms with susceptibility to hepatocellular carcinoma in a Han Chinese population[J]. DNA Cell Biol, 2011, 30(4): 229-234.

[271] Liu Y, Liu Y, Huang X, et al. Association of PvuII and XbaI polymorphisms in estrogen receptor alpha gene with the risk of hepatitis B virus infection in the Guangxi Zhuang population[J]. Infec Genet Evol, 2014, 27: 69-76.

[272] Peng Q, Yang S, Lao X, et al. Association of single nucleotide polymorphisms in VDR and DBP genes with HBV-related hepatocellular carcinoma risk in a Chinese population[J]. PLoS One, 2014, 9(12): e116026.

[273] Chen X, Zhang L, Chang Y, et al. Association of TNF-alpha genetic polymorphisms with hepatocellular carcinoma susceptibility: a case-control study in a Han Chinese population[J]. Int J Biol Markers, 2011, 26(3): 181-187.

[274] Liu P, Zhuo ZJ, Zhu Jh, et al. Association of TP53 rs1042522 C > G and miR-34b/c rs4938723 T > C polymorphisms with hepatoblastoma susceptibility: A seven-center case-control study[J]. J Gene Med, 2020, 22(7): e3182.

[275] Guo LY, Jin XP, Niu W, et al. Association of XPD and XRCC1 genetic polymorphisms with hepatocellular carcinoma risk[J]. Asian Pac J Cancer Prev, 2012, 13(9): 4423-4426.

[276] Xia WF, Ma XP, Li XR, et al. Association study of c.910A>G and c.1686C>G polymorphisms in XRCC1 gene with risk of hepatocellular carcinoma in the Chinese population[J]. Genet Mol Res, 2014, 13(1): 1314-1322.

[277] Wang R, Zhang J, Ma Y, et al. Association study of miR-149 rs2292832 and miR-608 rs4919510 and the risk of hepatocellular carcinoma in a large-scale population[J]. Mol Med Rep, 2014, 10(5): 2736-2744.

[278] Bi J, Zhong C, Li K, et al. Association study of single nucleotide polymorphisms in xrcc1 gene with risk of hepatocellular carcinoma in Chinese Han population[J]. BioMed Res Int, 2013, 2013: 138785.

[279] Hao YX, Wang JP, Zhao LF. Associations between three common MicroRNA polymorphisms and hepatocellular carcinoma risk in Chinese[J]. Asian Pac J Cancer Prev, 2014, 14(11): 6601-6604.

[280] Zhu J, Tan A, Yao Z, et al. Associations of toll-like receptor 4, 5 and 9 genetic variants with hepatitis b virus-related hepatocellular carcinoma and viral clearance in a guangxi male population[J]. Int J Clin Exp Med, 2017, 10(2): 3699-3705.

[281] Lai SW, Liao KF, Lin CL, et al. Case-control study examining the association between selective serotonin reuptake inhibitors use and hepatocellular carcinoma[J]. Front Pharmacol, 2017, 8(NOV): 861.

[282] Chao X, Jia Y, Feng X, et al. A Case–Control Study of ADCY9 Gene Polymorphisms and the Risk of Hepatocellular Carcinoma in the Chinese Han Population[J]. Front Oncol, 2020, 10: 1450.

[283] Liu TT, Fang Y, Xiong H, et al. A case-control study of the relationship between hepatitis B virus DNA level and risk of hepatocellular carcinoma in Qidong, China[J]. World J Gastroenterol, 2008, 14(19): 3059-3063.

[284] Deng B, Liu F, Luo L, et al. CASP 3 genetic polymorphisms and risk of Hepatocellular carcinoma: a case-control study in a Chinese population[J]. Tumour Biol, 2016, 37(7): 8985-8991.

[285] Pan X, Li M, Huang L, et al. CD44, IL-33, and ST2 Gene Polymorphisms on Hepatocellular Carcinoma Susceptibility in the Chinese Population[J]. BioMed Res Int, 2020, 2020: 2918517.

[286] Wang P, Peng J, Gong Y, et al. CDC25B is associated with the risk of hepatocellular carcinoma, but not related to persistent infection of hepatitis B virus in a Chinese population[J]. Mol Biol Rep, 2020, 47(5): 3361-3368.

[287] Yang Y, Xia T, Li N, et al. Combined effects of p53 and MDM2 polymorphisms on susceptibility and surgical prognosis in hepatitis B virus-related hepatocellular carcinoma[J]. Protein and Cell, 2013, 4(1): 71-81.

[288] Bei CH, Bai H, Yu HP, et al. Combined effects of six cytokine gene polymorphisms and SNP-SNP interactions on hepatocellular carcinoma risk in Southern Guangxi, China[J]. Asian Pac J Cancer Prev, 2014, 15(16): 6961-6967.

[289] Qu LS, Liu TT, Jin F, et al. Combined pre-S deletion and core promoter mutations related to hepatocellular carcinoma: A nested case-control study in China[J]. Hepatol Res, 2011, 41(1): 54-63.

[290] Li R, Shugart YY, Zhou W, et al. Common genetic variations of the cytochrome P450 1A1 gene and risk of hepatocellular carcinoma in a Chinese population[J]. Eur J Cancer, 2009, 45(7): 1239-1247.

[291] Zhang LH, Hao BB, Zhang CY, et al. Contributions of polymorphisms in mir146a, mir196a, and mir499 to the development of hepatocellular carcinoma[J]. Genet Mol Res, 2016, 15(3): gmr.15038582.

[292] Liu W, Ma N, Zhao D, et al. Correlation between the DEPDC5 rs1012068 polymorphism and the risk of HBV-related hepatocellular carcinoma[J]. Clin Res Hepatol Gastroenterol, 2019, 43(4): 446-450.

[293] Pan X, Wang G. Correlations of IL-23R gene polymorphism with clinicopathological characteristics and prognosis of hepatocellular carcinoma patients after interventional therapy[J]. Genomics, 2019, 111(4): 930-935.

[294] Xie Q, Chen Z, Xia L, et al. Correlations of PD-L1 gene polymorphisms with susceptibility and prognosis in hepatocellular carcinoma in a Chinese Han population[J]. Gene, 2018, 674: 188-194.

[295] Peng Q, Ren S, Lao X, et al. C-reactive protein genetic polymorphisms increase susceptibility to HBV-related hepatocellular carcinoma in a Chinese population[J]. Tumour Biol, 2014, 35(10): 10169-10176.

[296] Hu L, Liu J, Chen X, et al. CTLA-4 gene polymorphism +49 A/G contributes to genetic susceptibility to two infection-related cancers-hepatocellular carcinoma and cervical cancer[J]. Hum Immunol, 2010, 71(9): 888-891.

[297] Hu Z, Zhou Z, Xiong G, et al. Cyclin D1 G870A polymorphism and the risk of hepatocellular carcinoma in a Chinese population[J]. Tumour Biol, 2014, 35(6): 5607-5612.

[298] He J, Zhang Q, Ren Z, et al. Cyclooxygenase-2 -765 G/C polymorphisms and susceptibility to hepatitis B-related liver cancer in Han Chinese population[J]. Mol Biol Rep, 2012, 39(4): 4163-4168.

[299] Li X, Xu H, Gao P. Diabetes mellitus is a risk factor for hepatocellular carcinoma in patients with chronic hepatitis B virus infection in China[J]. Med Sci Monit, 2018, 24: 6729-6734.

[300] Zheng Z, Zhang C, Yan J, et al. Diabetes mellitus is associated with hepatocellular carcinoma: A retrospective case-control study in hepatitis endemic area[J]. PLoS One, 2013, 8(12): e84776.

[301] Hsieh YH, Chang WS, Tsai CW, et al. DNA double-strand break repair gene XRCC7 genotypes were associated with hepatocellular carcinoma risk in Taiwanese males and alcohol drinkers[J]. Tumour Biol, 2015, 36(6): 4101-4106.

[302] Wu Y, Lin JS. DNA methyltransferase 3B promoter polymorphism and its susceptibility to primary hepatocellular carcinoma in the Chinese Han nationality population: A case-control study[J]. World J Gastroenterol, 2007, 13(45): 6082-6086.

[303] Yang Z, Zhao J. Effect of APE1 and XRCC1 gene polymorphism on susceptibility to hepatocellular carcinoma and sensitivity to cisplatin[J]. Int J Clin Exp Med, 2015, 8(6): 9931-9936.

[304] Qu LS, Zhu J, Liu TT, et al. Effect of combined mutations in the enhancer II and basal core promoter of hepatitis B virus on development of hepatocellular carcinoma in Qidong, China[J]. Hepatol Res, 2014, 44(12): 1186-1195.

[305] Zhang Q, Ji XW, Hou XM, et al. Effect of functional nuclear factor-kappaB genetic polymorphisms on hepatitis B virus persistence and their interactions with viral mutations on the risk of hepatocellular carcinoma[J]. Ann Oncol, 2014, 25(12): 2413-2419.

[306] Wang B, Yeh CB, Lein MY, et al. Effects of HMGB1 Polymorphisms on the Susceptibility and Progression of Hepatocellular Carcinoma[J]. Int J Med Sci, 2016, 13(4): 304-309.

[307] Lin CH, Hsieh MJ, Lee HL, et al. Effects of MACC1 polymorphisms on hepatocellular carcinoma development and clinical characteristics[J]. J Cancer, 2020, 11(6): 1641-1647.

[308] Yip T, Wong G, Chan HLY, et al. Elevated testosterone increases risk of hepatocellular carcinoma in men with chronic hepatitis B and diabetes mellitus[J]. J Gastroenterol Hepatol, 2020, 35(12): 2210-2219.

[309] Niu J, Lin Y, Guo Z, et al. The Epidemiological Investigation on the Risk Factors of Hepatocellular Carcinoma: A Case-Control Study in Southeast China[J]. Medicine (Baltimore), 2016, 95(6): e2758.

[310] Chen K, Wei Y, Yang H, et al. Epidermal growth factor +61 G/A polymorphism and the risk of hepatocellular carcinoma in a Chinese population[J]. Gent Test and Mol Biomarkers, 2011, 15(4): 251-255.

[311] Li Y, Ou C, Shu H, et al. The ERCC1-4533/8092, TNF-α 238/308 polymorphisms and the risk of hepatocellular carcinoma in Guangxi Zhuang populations of China: Case-control study[J]. Medicine (Baltimore), 2016, 95(44): e5217.

[312] Zhai Y, Zhou G, Deng G, et al. Estrogen receptor alpha polymorphisms associated with susceptibility to hepatocellular carcinoma in hepatitis B virus carriers[J]. Gastroenterology, 2006, 130(7): 2001-2009.

[313] Zhang AY, Lai CL, Huang FY, et al. Evolutionary Changes of Hepatitis B Virus Pre-S Mutations Prior to Development of Hepatocellular Carcinoma[J]. PLoS One, 2015, 10(9): e0139478.

[314] Zhao B, Shen H, Liu F, et al. Exposure to organochlorine pesticides is an independent risk factor of hepatocellular carcinoma: A casecontrol study[J]. J Expos Sci Environ Epidemiol, 2012, 22(6): 541-548.

[315] Yin J, Wen J, Hang D, et al. Expression quantitative trait loci for CARD8 contributes to risk of two infection-related cancers - Hepatocellular carcinoma and cervical cancer[J]. PLoS One, 2015, 10(7): e0132352.

[316] Wang M, Liu X, Lin S, et al. FABP1 polymorphisms contribute to hepatocellular carcinoma susceptibility in Chinese population with liver cirrhosis: A case-control study[J]. J Cancer, 2018, 9(22): 4294-4300.

[317] Liu X, Baecker A, Wu M, et al. Family history of liver cancer may modify the association between HBV infection and liver cancer in a Chinese population[J]. Liver Int, 2019, 39(8): 1490-1503.

[318] Liao GS, Lo CH, Chen TW, et al. Fas/FasL polymorphisms are associated with hepatitis C related cirrhosis and serum Alpha-Fetoprotein with hepatocellular carcinoma patients[J]. J Cancer Res Pract, 2016, 3(1): 9-13.

[319] Sheu MJ, Hsieh MJ, Chiang WL, et al. Fibroblast growth factor receptor 4 polymorphism is associated with liver cirrhosis in hepatocarcinoma[J]. PLoS One, 2015, 10(4): e0122961.

[320] Cui LH, Song Y, Si H, et al. Folate metabolism-related gene polymorphisms and susceptibility to primary liver cancer in North China[J]. Med Oncol, 2012, 29(3): 1837-1842.

[321] Liu Y, Chai Y, Zhang J, et al. A Function Variant at miR-501 Alters Susceptibility to Hepatocellular Carcinoma in a Chinese Han Population[J]. Cell Physiol Biochem, 2016, 38(6): 2500-2508.

[322] Liu L, Zhou C, Zhou L, et al. Functional FEN1 genetic variants contribute to risk of hepatocellular carcinoma, esophageal cancer, gastric cancer and colorectal cancer[J]. Carcinogenesis, 2012, 33(1): 119-123.

[323] Yu Q, Zhou C, Wang J, et al. A functional insertion/deletion polymorphism in the promoter of PDCD6IP is associated with the susceptibility of hepatocellular carcinoma in a Chinese population[J]. DNA Cell Biol, 2013, 32(8): 451-457.

[324] Jiang L, Xu J, Ni J, et al. A functional insertion/deletion polymorphism in the proximal promoter of CD3G is associated with susceptibility for hepatocellular carcinoma in Chinese population[J]. DNA Cell Biol, 2012, 31(9): 1480-1485.

[325] Liu F, Luo L, Wei Y, et al. A functional NQO1 609C>T polymorphism and risk of hepatocellular carcinoma in a Chinese population[J]. Tumour Biol, 2013, 34(1): 47-53.

[326] Wang H, Song C, Qi Q, et al. Functional Polymorphisms in IRAKs Are Related to Hepatocellular Carcinoma Risk in Chinese Population[J]. BioMed Res Int, 2018, 2018: 1252849.

[327] Guo JC, Yang YJ, Zheng JF, et al. Functional rs6265 polymorphism in the brain-derived neurotrophic factor gene confers protection against neurocognitive dysfunction in posttraumatic stress disorder among Chinese patients with hepatocellular carcinoma[J]. J Cell Biochem, 2019, 120(6): 10434-10443.

[328] Jiang ZC, Tang XM, Zhao YR, et al. A functional variant at miR-34a binding site in toll-like receptor 4 gene alters susceptibility to hepatocellular carcinoma in a Chinese Han population[J]. Tumour Biol, 2014, 35(12): 12345-12352.

[329] Shen M, Lin L. Functional variants of autophagy-related genes are associated with the development of hepatocellular carcinoma[J]. Life Sci, 2019, 235: 116675.

[330] Chen CT, Liao WY, Hsu CC, et al. FUT2 genetic variants as predictors of tumor development with hepatocellular carcinoma[J]. Int J Med Sci, 2017, 14(9): 885-890.

[331] Zhou L, Zhang X, Chen X, et al. GC Glu416Asp and Thr420Lys polymorphisms contribute to gastrointestinal cancer susceptibility in a Chinese population[J]. Int J Clin Exp Med, 2011, 5(1): 72-79.

[332] Tian M, Zhao B, Martin FL, et al. Gene-environment interactions between GSTs polymorphisms and targeted epigenetic alterations in hepatocellular carcinoma following organochlorine pesticides (OCPs) exposure[J]. Environ Int, 2020, 134: 105313.

[333] Zhang S, Qiao K, Trieu C, et al. Genetic Polymorphism of Epidermal Growth Factor rs4444903 Influences Susceptibility to HCV-Related Liver Cirrhosis and Hepatocellular Carcinoma in a Chinese Han Population[J]. Clin Lab, 2017, 63(4): 845-850.

[334] Tang S, Yuan Y, He Y, et al. Genetic polymorphism of interleukin-6 influences susceptibility to HBV-related hepatocellular carcinoma in a male Chinese Han population[J]. Hum Immunol, 2014, 75(4): 297-301.

[335] Li S, Deng Y, Chen ZP, et al. Genetic polymorphism of interleukin-16 influences susceptibility to HBV-related hepatocellular carcinoma in a Chinese population[J]. Infec Genet Evol, 2011, 11(8): 2083-2088.

[336] Gao J, Xu HL, Gao S, et al. Genetic polymorphism of NFKB1 and NFKBIA genes and liver cancer risk: a nested case-control study in Shanghai, China[J]. BMJ Open, 2014, 4(2): e004427.

[337] Luo J, Chen S, Wang J, et al. Genetic polymorphisms in complement receptor 1 gene and its association with HBV-related liver disease: A case-control study[J]. Gene, 2019, 688: 107-118.

[338] Li R, Yang Y, An Y, et al. Genetic polymorphisms in DNA double-strand break repair genes XRCC5, XRCC6 and susceptibility to hepatocellular carcinoma[J]. Carcinogenesis, 2011, 32(4): 530-536.

[339] Yuan JM, Lu SC, Van Den Berg D, et al. Genetic polymorphisms in the Methylenetetrahydrofolate reductase and Thymidylate synthase genes and risk of hepatocellular carcinoma[J]. Hepatology, 2007, 46(3): 749-758.

[340] Huang X, Li H, Wang J, et al. Genetic polymorphisms in Toll-like receptor 3 gene are associated with the risk of hepatitis B virus-related liver diseases in a Chinese population[J]. Gene, 2015, 569(2): 218-224.

[341] Lao X, Ren S, Lu Y, et al. Genetic polymorphisms of C-reactive protein increase susceptibility to HBV-related hepatocellular carcinoma in a Guangxi male population[J]. Int J Clin Exp Pathol, 2015, 8(12): 16055-16063.

[342] Yuan JM, Fan Y, Ognjanovic S, et al. Genetic polymorphisms of epidermal growth factor in relation to risk of hepatocellular carcinoma: Two case-control studies[J]. BMC Gastroenterol, 2013, 13(1): 32.

[343] Jiang T, Cui L, Chen L, et al. Genetic polymorphisms of XRCC1 gene and susceptibility to hepatocellular carcinoma in Chinese population[J]. Med Oncol, 2014, 31(4): 887.

[344] Shi Y, Zhai W, Wang B, et al. Genetic susceptibility of eight nonsynonymous polymorphisms in HLA-DRB1 gene to hepatocellular carcinoma in Han Chinese[J]. Oncotarget, 2016, 7(49): 80935-80942.

[345] Zhong R, Liu L, Tian Y, et al. Genetic variant in SWI/SNF complexes influences hepatocellular carcinoma risk: a new clue for the contribution of chromatin remodeling in carcinogenesis[J]. Sci Rep, 2014, 4: 4147.

[346] Wu Z, Sun Y, Huang Y, et al. Genetic variant in visfatin gene promoter contributes to reduced risk of hepatocellular carcinoma in a Chinese population[J]. Oncotarget, 2016, 7(47): 77968-77977.

[347] Hu L, Zhai X, Liu J, et al. Genetic variants in human leukocyte antigen/DP-DQ influence both hepatitis B virus clearance and hepatocellular carcinoma development[J]. Hepatology, 2012, 55(5): 1426-1431.

[348] Tan A, Gao Y, Yao Z, et al. Genetic variants in IL12 influence both hepatitis B virus clearance and HBV-related hepatocellular carcinoma development in a Chinese male population[J]. Tumour Biol, 2016, 37(5): 6343-6348.

[349] Wei ZH, Li YY, Huang SQ, et al. Genetic variants in IL-33/ST2 pathway with the susceptibility to hepatocellular carcinoma in a Chinese population[J]. Cytokine, 2019, 118: 124-129.

[350] Liu L, Liu Y, Liu J, et al. Genetic variants in pseudogene E2F3P1 confer risk for HBV-related hepatocellular carcinoma in a Chinese population[J]. J Biomed Res, 2013, 27(3): 215-219.

[351] Ye X, Wang X, Shang L, et al. Genetic variants of ALDH2-rs671 and CYP2E1-rs2031920 contributed to risk of hepatocellular carcinoma susceptibility in a Chinese population[J]. Cancer Manage Res, 2018, 10: 1037-1050.

[352] Yuan R, Jiang C, Hong K, et al. Genetic variation in the Fat10 gene is associated with risk of hepatocellular carcinoma in a Chinese population[J]. Asian Pac J Cancer Prev, 2011, 12(8): 2117-2122.

[353] Huang MD, Chen XF, Xu G, et al. Genetic variation in the NBS1 gene is associated with hepatic cancer risk in a Chinese population[J]. DNA Cell Biol, 2012, 31(5): 678-682.

[354] Li D, Peng JJ, Tan Y, et al. Genetic variations in microRNA genes and susceptibility to hepatocellular carcinoma[J]. Genet Mol Res, 2015, 14(1): 1926-1931.

[355] Zhao X, Pan G, Yuan Q, et al. Genetic variations of CAV1 gene contribute to HCC risk: a case–control study[J]. Tumour Biol, 2014, 35(11): 11289-11293.

[356] Zhang H, Zhai Y, Hu Z, et al. Genome-wide association study identifies 1p36.22 as a new susceptibility locus for hepatocellular carcinoma in chronic hepatitis B virus carriers[J]. Nat Genet, 2010, 42(9): 755-758.

[357] Li Y, Zhai Y, Song Q, et al. Genome-Wide association study identifies a new locus at 7q21.13 associated with hepatitis b virus–Related hepatocellular carcinoma[J]. Clin Cancer Res, 2018, 24(4): 906-915.

[358] Chan HL, Hui AY, Wong ML, et al. Genotype C hepatitis B virus infection is associated with an increased risk of hepatocellular carcinoma[J]. Gut, 2004, 53(10): 1494-1498.

[359] Luo HC, Zhang HB, Xin XJ, et al. Haplotype-based case-control study of DNA repair gene XRCC3 and hepatocellular carcinoma risk in a Chinese population[J]. Tumour Biol, 2014, 35(4): 3415-3419.

[360] Li Y, Wang K, Dai L, et al. HapMap-based study of CIP2A gene polymorphisms and HCC susceptibility[J]. Oncol Lett, 2012, 4(2): 358-364.

[361] Lee MH, Yang HI, Lu SN, et al. Hepatitis C virus genotype 1b increases cumulative lifetime risk of hepatocellular carcinoma[J]. Int J Cancer, 2014, 135(5): 1119-1126.

[362] Wang XY, Fang AP, Chen PY, et al. High dietary inflammatory index scores are associated with an elevated risk of hepatocellular carcinoma in a case-control study[J]. Food Funct, 2018, 9(11): 5832-5842.

[363] Wong V, Yu J, Cheng A, et al. High serum interleukin-6 level predicts future hepatocellular carcinoma development in patients with chronic hepatitis B[J]. Int J Cancer, 2009, 124(12): 2766-2770.

[364] Zhou R-f, Chen X-l, Zhou Z-g, et al. Higher dietary intakes of choline and betaine are associated with a lower risk of primary liver cancer: a case-control study[J]. Sci Rep, 2017, 7(679):

[365] Qi JH, Wang J, Chen J, et al. High-resolution melting analysis reveals genetic polymorphisms in MicroRNAs confer hepatocellular carcinoma risk in Chinese patients[J]. BMC Cancer, 2014, 14(1): 643.

[366] Zhang C, Tian YP, Wang Y, et al. hTERT rs2736098 genetic variants and susceptibility of hepatocellular carcinoma in the Chinese population: A case-control study[J]. Hepatobiliary Pancreatic Dis Int, 2013, 12(1): 74-79.

[367] Ma Y, Wang R, Zhang J, et al. Identification of miR-423 and miR-499 Polymorphisms on Affecting the Risk of Hepatocellular Carcinoma in a Large-Scale Population[J]. Gent Test and Mol Biomarkers, 2014, 18(7): 516-524.

[368] Liu L, Xu Y, Liu Z, et al. IL12 polymorphisms, HBV infection and risk of hepatocellular carcinoma in a high-risk Chinese population[J]. Int J Cancer, 2011, 128(7): 1692-1696.

[369] Zhou J, Liao W, Zhao Y, et al. IL-10 and IL-10RB gene polymorphisms are correlated with hepatitis B-related hepatocellular carcinoma in the Chinese Han population[J]. Transl Cancer Res, 2017, 6(2): 432-440.

[370] Wu W, Zeng Y, Lin J, et al. IL-17 and IL-21 polymorphisms in relation to HBV related hepatocellular carcinoma in Chinese Han population[J]. Infect Genet Evol, 2020, 87: 104638-104638.

[371] Dai ZJ, Liu XH, Wang M, et al. IL-18 polymorphisms contribute to hepatitis B virus-related cirrhosis and hepatocellular carcinoma susceptibility in Chinese population: A case-control study[J]. Oncotarget, 2017, 8(46): 81350-81360.

[372] Shiu JS, Hsieh MJ, Chiou HL, et al. Impact of ADAM10 gene polymorphisms on hepatocellular carcinoma development and clinical characteristics[J]. Int J Med Sci, 2018, 15(12): 1334-1340.

[373] Lin Y, Su C, Niu J, et al. Impact of mannose-binding lectin 2 polymorphism on the risk of hepatocellular carcinoma: a case-control study in Chinese Han population[J]. J Epidemiol, 2015, 25(5): 387-391.

[374] Wang B, Hsu CJ, Lee HL, et al. Impact of matrix metalloproteinase-11 gene polymorphisms upon the development and progression of hepatocellular carcinoma[J]. Int J Med Sci, 2018, 15(6): 653-658.

[375] Wang X, Hao X, Wang H, et al. Impact of NFKB1 and NFKBIA gene polymorphism and additional gene-gene interaction on liver cancer risk in Chinese population[J]. Int J Clin Exp Pathol, 2016, 9(12): 12968-12975.

[376] Wang B, Chou YE, Lien MY, et al. Impacts of CCL4 gene polymorphisms on hepatocellular carcinoma susceptibility and development[J]. Int J Med Sci, 2017, 14(9): 880-884.

[377] Wu JS, Chen YP, Wang LC, et al. Implication of polymorphisms in DNA repair genes with an increased risk of hepatocellular carcinoma[J]. Genet Mol Res, 2014, 13(2): 3812-3818.

[378] Yu Q, Qian W, Wang J, et al. An indel polymorphism in the 3' untranslated region of JAK1 confers risk for hepatocellular carcinoma possibly by regulating JAK1 transcriptional activity in a Chinese population[J]. Oncol Lett, 2018, 15(5): 8088-8094.

[379] Wang J, Zhu J, Mao DH, et al. An indel polymorphism in the 3’ untranslated region of hmgb1 confers risk for hepatocellular carcinoma by regulating hmgb1 transcriptional activity in a chinese population[J]. Neoplasma, 2020, 67(1): 61-67.

[380] Wang C, Li L, Yin Z, et al. An indel polymorphism within pre-miR3131 confers risk for hepatocellular carcinoma[J]. Carcinogenesis, 2017, 38(2): 168-176.

[381] Zeng Z, Tu J, Cheng J, et al. Influence of CCND1 G870A polymorphism on the risk of HBV-related HCC and cyclin D1 splicing variant expression in Chinese population[J]. Tumour Biol, 2015, 36(9): 6891-6900.

[382] Gao Y, He Y, Ding J, et al. An insertion/deletion polymorphism at miRNA-122-binding site in the interleukin-1alpha 3' untranslated region confers risk for hepatocellular carcinoma[J]. Carcinogenesis, 2009, 30(12): 2064-2069.

[383] Zhu Z, Jiang Y, Chen S, et al. An insertion/deletion polymorphism in the 3' untranslated region of type I collagen a2 (COL1A2) is associated with susceptibility for hepatocellular carcinoma in a Chinese population[J]. Cancer Genet, 2011, 204(5): 265-269.

[384] Chen S, He Y, Ding J, et al. An insertion/deletion polymorphism in the 3′ untranslated region of β-transducin repeat-containing protein (βTrCP) is associated with susceptibility for hepatocellular carcinoma in Chinese[J]. Biochem Biophys Res Commun, 2010, 391(1): 552-556.

[385] Zhu Z, Gao X, He Y, et al. An insertion/deletion polymorphism within RERT-lncRNA modulates hepatocellular carcinoma risk[J]. Cancer Res, 2012, 72(23): 6163-6172.

[386] Gao X, Huang M, Liu L, et al. Insertion/Deletion Polymorphisms in the Promoter Region of BRM Contribute to Risk of Hepatocellular Carcinoma in Chinese Populations[J]. PLoS One, 2013, 8(1): e55169.

[387] Shen N, Gong J, Wang Y, et al. Integrative genomic analysis identifies that SERPINA6-rs1998056 Regulated by FOXA/ERα is associated with female hepatocellular carcinoma[J]. PLoS One, 2014, 9(9): e107246.

[388] Qiu M, Liu Y, Yu X, et al. Interaction between p53 codon 72 and MDM2 309T>G polymorphisms and the risk of hepatocellular carcinoma[J]. Tumour Biol, 2016, 37(3): 3863-3870.

[389] Li H, Liu F, Zhu H, et al. Interaction between polymorphisms of IFN-γ and MICA correlated with hepatocellular carcinoma[J]. Med Sci Monit, 2016, 22: 549-553.

[390] Yao JG, Huang XY, Long XD. Interaction of DNA repair gene polymorphisms and aflatoxin B1 in the risk of hepatocellular carcinoma[J]. Int J Clin Exp Pathol, 2014, 7(9): 6231-6244.

[391] Xie J, Zhang Y, Zhang Q, et al. Interaction of signal transducer and activator of transcription 3 polymorphisms with hepatitis B virus mutations in hepatocellular carcinoma[J]. Hepatology, 2013, 57(6): 2369-2377.

[392] Hou Y, Zhang Y, Qin L, et al. Interferon-induced transmembrane protein-3 rs12252-CC is associated with low differentiation and progression of hepatocellular carcinoma[J]. Medicine, 2019, 98(2): e13996.

[393] Yao JY, Chao K, Li MR, et al. Interleukin-21 gene polymorphisms and chronic hepatitis B infection in a Chinese population[J]. World J Gastroenterol, 2015, 21(14): 4232-4239.

[394] Liu T, Song J, Zhang M, et al. Interleukin-21 receptor gene polymorphism is associated with hepatitis B virus-related hepatocellular carcinoma in Chinese patients[J]. J Clin Lab Anal, 2019, 33(5): e22860.

[395] Sopipong W, Tangkijvanich P, Payungporn S, et al. The KIF1B (rs17401966) single nucleotide polymorphism is not associated with the development of HBV-related hepatocellular carcinoma in Thai patients[J]. Asian Pac J Cancer Prev, 2013, 14(5): 2865-2869.

[396] Wang L, Rasul A, Liu Z, et al. The Loss of Masculine with Declined Serum DHT Is Associated With High Risk of Hepatocellular Carcinoma in Chinese Men[J]. Front Endocrinol (Lausanne), 2020, 11: 362.

[397] Wang PS, Kuai J, Li H, et al. Mannose-binding lectin 2 rs11003123 polymorphism is associated with the development of hepatocellular carcinoma in patients with hepatitis B-related cirrhosis in the Chinese population[J]. Hepatobiliary Pancreatic Dis Int, 2016, 15(3): 282-288.

[398] Mu LN, Cao W, Zhang ZF, et al. Methylenetetrahydrofolate reductase (MTHFR) C677T and A1298C polymorphisms and the risk of primary Hepatocellular Carcinoma (HCC) in a Chinese population[J]. Cancer Causes and Control, 2007, 18(6): 665-675.

[399] Chao X, Feng X, Shi H, et al. MIR17HG polymorphism (rs7318578) is associated with liver cancer risk in the Chinese Han population[J]. Biosci Rep, 2020, 40(8): BSR20193312.

[400] Cong N, Chen H, Bu WZ, et al. miR-146a G>C polymorphisms and risk of hepatocellular carcinoma in a Chinese population[J]. Tumour Biol, 2014, 35(6): 5669-5673.

[401] Huang Y, Sheng S, Chen B, et al. MiR-146a genetic polymorphism contributes to the susceptibility to hepatocellular carcinoma in a Chinese population[J]. Int J Clin Exp Pathol, 2017, 10(2): 1833-1839.

[402] Ji J, Xu M, Tu J, et al. MiR-155 and its functional variant rs767649 contribute to the susceptibility and survival of Hepatocellular carcinoma[J]. Oncotarget, 2016, 7(37): 60303-60309.

[403] Qiu M, Liu Y, Lin Q, et al. A miR-182 variant and risk of hepatocellular carcinoma in a southern Chinese population[J]. Hum Genomics, 2020, 14(1): 38.

[404] Shan YF, Huang YH, Chen ZK, et al. miR-499A>G rs3746444 and miR-146aG>C expression and hepatocellular carcinoma risk in the Chinese population[J]. Genet Mol Res, 2013, 12(4): 5365-5371.

[405] Wu X, Xin Z, Zhang W, et al. A missense polymorphism in ATF6 gene is associated with susceptibility to hepatocellular carcinoma probably by altering ATF6 level[J]. Int J Cancer, 2014, 135(1): 61-68.

[406] Jiao X, Luo Y, Yang B, et al. The MTHFR C677T mutation is not a risk factor recognized for HBV-related HCC in a population with a high prevalence of this genetic marker[J]. Infec Genet Evol, 2017, 49: 66-72.

[407] Wang D, Qi X, Liu F, et al. A multicenter matched case-control analysis on seven polymorphisms from HMGB1 and RAGE genes in predicting hepatocellular carcinoma risk[J]. Oncotarget, 2017, 8(30): 50109-50116.

[408] Qi P, Wang H, Chen YM, et al. No association of EGF 5'UTR variant A61G and hepatocellular carcinoma in Chinese patients with chronic hepatitis B virus infection[J]. Pathology, 2009, 41(6): 555-560.

[409] Wan J, Huang M, Zhao H, et al. A novel tetranucleotide repeat polymorphism within KCNQ1OT1 confers risk for hepatocellular carcinoma[J]. DNA Cell Biol, 2013, 32(11): 628-634.

[410] Huang L, Mo Z, Lao X, et al. PIN1 genetic polymorphisms and the susceptibility of HBV-related hepatocellular carcinoma in a Guangxi population[J]. Tumour Biol, 2016, 37(5): 6599-6606.

[411] Chen CL, Yang WS, Yang HI, et al. Plasma adipokines and risk of hepatocellular carcinoma in chronic hepatitis B virus-infected carriers: A prospective study in Taiwan[J]. Cancer Epidemiol Biomarkers Prev, 2014, 23(8): 1659-1671.

[412] Long XD, Ma Y, Zhou YF, et al. Polymorphism in xeroderma pigmentosum complementation group C codon 939 and aflatoxin B1-related hepatocellular carcinoma in the Guangxi population[J]. Hepatology, 2010, 52(4): 1301-1309.

[413] Pan HZ, Liang J, Yu Z, et al. Polymorphism of DNA repair gene XRCC1 and hepatocellular carcinoma risk in Chinese population[J]. Asian Pac J Cancer Prev, 2011, 12(11): 2947-2950.

[414] Yu Q, Zhou CX, Chen NS, et al. A polymorphism within ErbB4 is associated with risk for hepatocellular carcinoma in Chinese population[J]. World J Gastroenterol, 2012, 18(4): 383-387.

[415] Xin Z, Zhang W, Xu A, et al. Polymorphisms in the potential functional regions of the TGF-β 1 and TGF-β receptor genes and disease susceptibility in HBV-related hepatocellular carcinoma patients[J]. Mol Carcinog, 2012, 51(SUPPL. 1): E123-E131.

[416] Yuan T, Wei J, Luo J, et al. Polymorphisms of base-excision repair genes hOGG1 326cys and XRCC1 280His increase hepatocellular carcinoma risk[J]. Dig Dis Sci, 2012, 57(9): 2451-2457.

[417] Liu F, Luo LM, Wei YG, et al. Polymorphisms of the CYP1B1 gene and hepatocellular carcinoma risk in a Chinese population[J]. Gene, 2015, 564(1): 14-20.

[418] Jia ZF, Su HY, Li XL, et al. Polymorphisms of UGT1A7 and XRCC1 are associated with an increased risk of hepatocellular carcinoma in Northeast China[J]. Chin J Cancer Res, 2010, 22(4): 260-266.

[419] Zou HZ, Zhao YQ. Positive association between miR-499a>G and hepatocellular carcinoma risk in a chinese population[J]. Asian Pac J Cancer Prev, 2013, 14(3): 1769-1772.

[420] Xu Y, Liu L, Liu J, et al. A potentially functional polymorphism in the promoter region of miR-34b/c is associated with an increased risk for primary hepatocellular carcinoma[J]. Int J Cancer, 2011, 128(2): 412-417.

[421] Liu L, Tian N, Zhou C, et al. A potentially functional variant of ARID1B interacts with physical activity in association with risk of hepatocellular carcinoma[J]. Oncotarget, 2017, 8(19): 31057-31064.

[422] Li CG, Zhao ZM, Hu MG, et al. Predictive role of glutathione-s-transferase gene polymorphisms in risk and prognosis of hepatocellular carcinoma[J]. Asian Pac J Cancer Prev, 2012, 13(7): 3247-3252.

[423] Yan P, Xia M, Gao F, et al. Predictive role of miR-146a rs2910164 (C>G), miR-149 rs2292832 (T>C), miR-196a2 rs11614913 (T>C) and miR-499 rs3746444 (T>C) in the development of hepatocellular carcinoma[J]. Int J Clin Exp Pathol, 2015, 8(11): 15177-15183.

[424] Wu J, Lv S, An J, et al. Pre-miR-149 rs71428439 polymorphism is associated with increased cancer risk and AKT1/cyclinD1 signaling in hepatocellular carcinoma[J]. Int J Clin Exp Med, 2015, 8(8): 13628-13633.

[425] Qu L, Kuai X, Liu T, et al. Pre-S deletion and complex mutations of hepatitis B virus related to young age hepatocellular carcinoma in Qidong, China[J]. PLoS One, 2013, 8(3): e59583.

[426] Liu CJ, Ma XW, Zhang XJ, et al. pri-miR-34b/c rs4938723 polymorphism is associated with hepatocellular carcinoma risk: A case-control study in a Chinese population[J]. Int J Med Sci, 2017, 8(1): 1-7.

[427] Yuan T, Deng S, Liu H, et al. Relationship between XRCC1 and XPD polymorphisms and the risk of the development of hepatocellular carcinoma: A case-control study[J]. Exp Ther Med, 2012, 4(2): 285-290.

[428] Chien MH, Yeh CB, Li YC, et al. Relationship of interleukin-8 gene polymorphisms with hepatocellular carcinoma susceptibility and pathological development[J]. J Surg Oncol, 2011, 104(7): 798-803.

[429] Nan YL, Hu YL, Liu ZK, et al. Relationships between cell cycle pathway gene polymorphisms and risk of hepatocellular carcinoma[J]. World J Gastroenterol, 2016, 22(24): 5558-5567.

[430] Liu Y, Huang L, Lu Y, et al. Relationships between the Osteocalcin gene polymorphisms, serum osteocalcin levels, and hepatitis B virus-related hepatocellular carcinoma in a Chinese population[J]. PLoS One, 2015, 10(1): e0116479.

[431] Chen K, Shi W, Xin Z, et al. Replication of Genome Wide Association Studies on Hepatocellular Carcinoma Susceptibility Loci in a Chinese Population[J]. PLoS One, 2013, 8(10): e77315.

[432] Chen W, Wang M, Zhang Z, et al. Replication the association of 2q32.2-q32.3 and 14q32.11 with hepatocellular carcinoma[J]. Gene, 2015, 561(1): 63-67.

[433] Jia ZF, Su M, He M, et al. Risk analysis of hepatocellular carcinoma in Northeast China[J]. Chin J Cancer Res, 2009, 21(4): 304-309.

[434] Gao YH, Li QQ, Wang CG, et al. The role of IL22 polymorphisms on liver cirrhosis in patients with hepatitis B virus: A case control study[J]. Medicine (Baltimore), 2019, 98(44): e17867.

[435] Peng MW, Lu SQ, Liu J, et al. Role of IL-10 polymorphisms in susceptibility to hepatitis B virus-related hepatocellular carcinoma[J]. Genet Mol Res, 2016, 15(1): gmr7984.

[436] Li Z, Yuan W, Ning S, et al. Role of Leptin Receptor (LEPR) Gene Polymorphisms and Haplotypes in Susceptibility to Hepatocellular Carcinoma in Subjects with Chronic Hepatitis B Virus Infection[J]. Mol Diagn Ther, 2012, 16(6): 383-388.

[437] Liu MF, Chen WQ, He YZ, et al. Role of miR-149C>T polymorphisms on the risk of hepatocellular carcinoma in aChinese population[J]. Genet Mol Res, 2014, 13(3): 7184-7189.

[438] Zhao X, Wang T, Liu B, et al. Significant association between upstream transcription factor 1 rs2516839 polymorphism and hepatocellular carcinoma risk: a case-control study[J]. Tumour Biol, 2015, 36(4): 2551-2558.

[439] Cui M, Chen Q, He C, et al. A single nucleotide polymorphism CTSB rs12898 is associated with primary hepatic cancer in a Chinese population[J]. Int J Clin Exp Pathol, 2019, 12(8): 3063-3069.

[440] Chang SC, Chang PY, Butler B, et al. Single nucleotide polymorphisms of one-carbon metabolism and cancers of the esophagus, stomach, and liver in a Chinese population[J]. PLoS One, 2014, 9(10): e109235.

[441] Li J, Cheng G, Wang S. A Single-Nucleotide Polymorphism of miR-196a2T>C rs11614913 Is Associated with Hepatocellular Carcinoma in the Chinese Population[J]. Gent Test and Mol Biomarkers, 2016, 20(4): 213-215.

[442] Ji J, Xu M, Zhao Z, et al. SMAD7 loci contribute to risk of hepatocellular carcinoma and clinicopathologic development among Chinese Han population[J]. Oncotarget, 2016, 7(16): 22186-22192.

[443] Jiang BG, Yang Y, Liu H, et al. SOCS3 genetic polymorphism is associated with clinical features and prognosis of hepatocellular Carcinoma patients receiving hepatectomy[J]. Medicine (United States), 2015, 94(40): e1344.

[444] Lai SW, Liao KF, Lai HC, et al. Statin use and risk of hepatocellular carcinoma[J]. Eur J Epidemiol, 2013, 28(6): 485-492.

[445] Han X, Xing Q, Li Y, et al. Study on the DNA Repair Gene XRCC1 and XRCC3 Polymorphism in Prediction and Prognosis of Hepatocellular Carcinoma Risk[J]. Hepato-gastroenterology, 2012, 59(119): 2285-2289.

[446] Wan PQ, Wu JZ, Huang LY, et al. TGF-beta 1 polymorphisms and familial aggregation of liver cancer in Guangxi, China[J]. Genet Mol Res, 2015, 14(3): 8147-8160.

[447] Shi ZY, Du CY. Tumor necrosis factor alpha 308 G/A polymorphism and hepatocellular carcinoma risk in a Chinese population[J]. Gent Test and Mol Biomarkers, 2011, 15(7): 569-572.

[448] Feng H, Kuai J, Zhang M, et al. Tumor necrosis factor-alpha gene -308G > A polymorphism alters the risk of hepatocellular carcinoma in a Han Chinese population[J]. Diagn Pathol, 2014, 9: 199.

[449] Yang MD, Hsu CM, Chang WS, et al. Tumor Necrosis Factor-α Genotypes Are Associated with Hepatocellular Carcinoma Risk in Taiwanese Males, Smokers and Alcohol Drinkers[J]. Anticancer Res, 2015, 35(10): 5417-5423.

[450] Li XD, Li ZG, Song XX, et al. A variant in microRNA-196a2 is associated with susceptibility to hepatocellular carcinoma in Chinese patients with cirrhosis[J]. Pathology, 2010, 42(7): 669-673.

[451] Wang B, Hsu CJ, Chou CH, et al. Variations in the AURKA gene: Biomarkers for the development and progression of hepatocellular carcinoma[J]. Int J Med Sci, 2018, 15(2): 170-175.

[452] He Y, Ni J, Chen S, et al. The vascular endothelial growth factor-2549 insertion/deletion polymorphism is not associated with susceptibility to hepatocellular carcinoma in Chinese[J]. DNA Cell Biol, 2010, 29(7): 393-396.

[453] Long XD, Ma Y, Wei YP, et al. X-ray repair cross-complementing group 1 (XRCC1) Arg 399 Gln polymorphism and aflatoxin B1 (AFB1)-related hepatocellular carcinoma (HCC) in Guangxi population[J]. Chin J Cancer Res, 2005, 17(1): 17-21.
