# Supplementary material for: A polygenetic risk score combined with environmental factors better predict susceptibility to hepatocellular carcinoma in Chinese population
Source: Cancer Med. 2024 May 2;13(9):e7230. doi: 10.1002/cam4.7230 (PMC11066500; doi:10.1002/cam4.7230)
Supplement: Supplementary file 2 — Data S2. [file CAM4-13-e7230-s002.docx]

Table S1. Quantitative combination of environmental factors and HCC

| Factors | No. of studies | HCC | Control | *OR* (95%*CI*) | *P* |
| --- | --- | --- | --- | --- | --- |
| HBV infection | 116 |  |  |  |  |
| No |  | 18,759 | 66,492 | 1 |  |
| Yes |  | 35,154 | 9,070 | 14.35(12.45,16.54) | <0.001^r^ |
| HCV infection | 24 |  |  |  |  |
| No |  | 10,342 | 27,791 | 1 |  |
| Yes |  | 2,238 | 697 | 5.40(3.53,8.27) | <0.001^r^ |
| Smoking history | 308 |  |  |  |  |
| No |  | 72,913 | 104,960 | 1 |  |
| Yes |  | 60,298 | 67,508 | 1.40(1.32,1.48) | <0.001^r^ |
| Drinking history | 315 |  |  |  |  |
| No |  | 74,975 | 113,937 | 1 |  |
| Yes |  | 58,391 | 60,886 | 1.61(1.52,1.71) | <0.001^r^ |
| Hypertension | 27 |  |  |  |  |
| No |  | 34,031 | 258,732 | 1 |  |
| Yes |  | 11,412 | 67,576 | 1.01(0.93,1.10) | 0.800^r^ |
| T2DM | 49 |  |  |  |  |
| No |  | 48,388 | 331,138 | 1 |  |
| Yes |  | 8,637 | 25,388 | 1.64(1.41,1.91) | <0.001^r^ |
| Blood type | 3 |  |  |  |  |
| No |  | 1,311 | 4,169 | 1 |  |
| Yes |  | 511 | 1,738 | 0.74(0.50,1.10) | 0.133^r^ |
| Cirrhosis | 21 |  |  |  |  |
| No |  | 30,768 | 302,932 | 1 |  |
| Yes |  | 3,521 | 3,932 | 6.53(4.79,8.90) | <0.001^r^ |
| Fatty liver | 21 |  |  |  |  |
| No |  | 3,260 | 21,093 | 1 |  |
| Yes |  | 6,298 | 3,644 | 5.57(2.90,10.68) | <0.001^r^ |
| Family history of HBV | 12 |  |  |  |  |
| No |  | 2,154 | 2,463 | 1 |  |
| Yes |  | 903 | 619 | 1.63(1.21,2.19) | 0.001^r^ |
| Family history of HCC | 57 |  |  |  |  |
| No |  | 18,132 | 26,890 | 1 |  |
| Yes |  | 3,222 | 1,633 | 3.32(2.78,3.97) | <0.001^r^ |
| Family history of cancer | 34 |  |  |  |  |
| No |  | 13,043 | 22,771 | 1 |  |
| Yes |  | 2,478 | 1591 | 2.57(2.09,3.16) | <0.001^r^ |

HBV, hepatitis B virus; HCV, hepatitis C virus; HCC, hepatocellular carcinoma; T2DM, type 2 diabetes mellitus; *OR*, odd ratios; *CI*, confidence intervals. ^f^, fixed model; ^r^, random model

Table S1 (Continued). Quantitative combination of environmental factors and HCC

| Factors | No. of studies | | HCC | Control | *OR* (95%*CI*) | *P* |
| --- | --- | --- | --- | --- | --- | --- |
| Aflatoxin-contaminated food | 5 |  | |  |  |  |
| No |  | 718 | | 897 | 1 |  |
| Yes |  | 210 | | 123 | 2.28(1.37,3.80) | 0.002^r^ |
| Fried and smoked food | 7 |  | |  |  |  |
| No |  | 1,225 | | 1,414 | 1 |  |
| Yes |  | 566 | | 410 | 1.44(1.31,1.58) | <0.001^f^ |
| Sashimi | 4 |  | |  |  |  |
| No |  | 1,323 | | 1,648 | 1 |  |
| Yes |  | 554 | | 246 | 2.34(0.86,6.32) | 0.095^r^ |

HBV, hepatitis B virus; HCV, hepatitis C virus; HCC, hepatocellular carcinoma; T2DM, type 2 diabetes mellitus; *OR*, odd ratios; *CI*, confidence intervals. ^f^, fixed model; ^r^, random model

Table S2. Quantitative combination of genetic factors and HCC

| Gene | SNPs | Per-allele | | Heterozygous | | Homozygous | | Dominant model | | Recessive model | |
| --- | --- | --- | --- | --- | --- | --- | --- | --- | --- | --- | --- |
|  |  | *OR* (95%*CI*) | *P* | *OR* (95%*CI*) | *P* | *OR* (95%*CI*) | *P* | *OR* (95%*CI*) | *P* | *OR* (95%*CI*) | *P* |
| **Related to inflammation and immune response** | | | | | | | | | | | |
| *COX-2* | rs5275 | 1.07(0.96,1.19) | 0.219^f^ | 1.00(0.89,1.12) | 0.980^f^ | 1.47(1.03,2.11) | 0.036^f^ | 1.03(0.93,1.14) | 0.571^f^ | 1.48(1.03,2.13) | 0.036^f^ |
| *COX-2* | rs689466 | 1.19(1.01,1.39) | 0.034^r^ | 1.05(0.99,1.10) | 0.094^f^ | 1.42(1.02,1.96) | 0.037^r^ | 1.27(1.00,1.61) | 0.055^r^ | 1.24(0.99,1.55) | 0.066^r^ |
| *CTLA-4* | rs231775 | 1.19(0.83,1.70) | 0.345^r^ | 1.06(0.64,1.76) | 0.821^r^ | 1.35(0.64,2.86) | 0.435^r^ | 1.20(0.65,2.24) | 0.558^r^ | 1.23(0.83,1.82) | 0.308^r^ |
| *IL-6* | rs1800796 | 1.08(1.00,1.17) | 0.046^f^ | 1.07(0.99,1.17) | 0.092^f^ | 1.17(0.91,1.51) | 0.219^f^ | 1.08(1.00,1.16) | 0.054^f^ | 1.14(0.89,1.47) | 0.308^f^ |
| *IL-8* | rs4073 | 1.12(1.04,1.20) | 0.003^f^ | 1.51(1.01,2.26) | 0.045^r^ | 1.17(0.98,1.40) | 0.082^f^ | 1.47(1.06,2.06) | 0.022^r^ | 1.01(0.64,1.58) | 0.979^r^ |
| *IL-10* | rs1800871 | 1.11(0.91,1.35) | 0.291^r^ | 1.13(0.88,1.46) | 0.342^r^ | 1.19(0.77,1.84) | 0.436^r^ | 1.15(0.87,1.52) | 0.329^r^ | 1.16(0.89,1.50) | 0.283^r^ |
| *IL-10* | rs1800872 | 1.11(1.04,1.18) | 0.003^f^ | 1.09(1.02,1.17) | 0.019^f^ | 1.40(0.98,2.01) | 0.069^r^ | 1.09(1.03,1.16) | 0.004^f^ | 1.27(0.88,1.82) | 0.198^r^ |
| *IL-12B* | rs3212227 | 1.02(0.97,1.06) | 0.466^f^ | 1.03(0.98,1.08) | 0.217^f^ | 1.03(0.94,1.13) | 0.547^f^ | 1.02(0.99,1.06) | 0.244^f^ | 1.00(0.90,1.11) | 0.964^f^ |
| *IL-16* | rs4072111 | 1.03(0.90,1.17) | 0.706^f^ | 0.94(0.82,1.07) | 0.339^f^ | 1.39(0.93,2.06) | 0.105^f^ | 0.98(0.87,1.10) | 0.682^f^ | 1.47(0.98,2.20) | 0.063^f^ |
| *IL-18* | rs187238 | 1.09(0.69,1.72) | 0.716^r^ | 0.96(0.91,1.01) | 0.108^f^ | 0.99(0.98,1.01) | 0.443^f^ | 0.99(0.98,1.01) | 0.274^f^ | 1.18(0.68,2.03) | 0.565^r^ |
| *IL-18* | rs1946518 | 1.01(0.96,1.07) | 0.659^f^ | 1.01(0.95,1.07) | 0.872^f^ | 1.02(0.92,1.14) | 0.671^f^ | 1.01(0.96,1.05) | 0.770^f^ | 1.03(0.90,1.17) | 0.668^f^ |
| *IL-21* | rs2221903 | 1.01(0.99,1.03) | 0.554^f^ | 1.00(0.96,1.05) | 0.862^f^ | 1.00(0.99,1.02) | 0.748^f^ | 1.00(0.99,1.01) | 0.770^f^ | 1.01(0.97,1.05) | 0.573^f^ |
| *IFNG* | rs2069705 | 1.01(0.94,1.09) | 0.741^f^ | 0.96(0.89,1.04) | 0.302^f^ | 1.10(0.92,1.32) | 0.287^f^ | 0.98(0.92,1.05) | 0.638^f^ | 1.15(0.95,1.39) | 0.147^f^ |
| *MICA* | rs2596542 | 1.10(0.80,1.52) | 0.550^r^ | 1.05(0.95,1.17) | 0.314^f^ | 1.23(0.63,2.40) | 0.555^r^ | 1.12(0.78,1.60) | 0.539^r^ | 1.15(0.69,1.91) | 0.588^r^ |
| *TNF-α* | rs1799964 | 1.10(1.00,1.20) | 0.045^f^ | 1.01(0.92,1.11) | 0.850^f^ | 1.58(1.18,2.12) | 0.002^f^ | 1.05(0.96,1.14) | 0.290^f^ | 1.60(1.19,2.15) | 0.002^f^ |
| *TLR4* | rs11536889 | 1.13(0.96,1.32) | 0.154^f^ | 1.07(0.97,1.17) | 0.173^f^ | 1.29(0.99,1.69) | 0.060^f^ | 1.08(0.99,1.17) | 0.078^f^ | 1.25(0.95,1.65) | 0.118^f^ |
| **Related to DNA synthesis and damage repair** | | | | | | | | | | | |
| *GSTP1* | rs1695 | 1.09(0.98,1.21) | 0.124^f^ | 1.00(0.90,1.12) | 0.957^f^ | 1.35(1.02,1.80) | 0.039^f^ | 1.03(0.94,1.14) | 0.498^f^ | 1.40(1.04,1.89) | 0.027^f^ |

SNPs, single nucleotide polymorphisms; *OR*, odd ratios; *CI*, confidence intervals. ^f^, fixed model; ^r^, random model

Table S2 (Continued). Quantitative combination of genetic factors and HCC

| Gene | SNPs | Per-allele | | Heterozygous | | Homozygous | | Dominant model | | Recessive model | |
| --- | --- | --- | --- | --- | --- | --- | --- | --- | --- | --- | --- |
|  |  | *OR* (95%*CI*) | *P* | *OR* (95%*CI*) | *P* | *OR* (95%*CI*) | *P* | *OR* (95%*CI*) | *P* | *OR* (95%*CI*) | *P* |
| *hOGG1* | rs1052133 | 1.42(1.14,1.77) | 0.002^r^ | 1.24(1.14,1.36) | <0.001^f^ | 2.05(1.27,3.32) | 0.003^r^ | 1.87(1.32,2.67) | <0.001^r^ | 1.31(1.04,1.65) | 0.022^f^ |
| *MDM2* | rs2279744 | 1.20(1.02,1.40) | 0.027^r^ | 1.27(1.00,1.61) | 0.054^r^ | 1.44(1.05,1.98) | 0.024^r^ | 1.33(1.03,1.73) | 0.030^r^ | 1.22(1.01,1.47) | 0.041^r^ |
| *TP53* | rs1042522 | 1.17(1.02,1.36) | 0.030^r^ | 1.03(1.00,1.06) | 0.043^f^ | 1.37(1.02,1.84) | 0.040^r^ | 1.19(1.02,1.4) | 0.031^r^ | 1.26(0.99,1.61) | 0.062^r^ |
| *XPC* | rs2228001 | 1.31(1.11,1.53) | 0.001^r^ | 1.15(1.09,1.20) | <0.001^f^ | 1.72(1.28,2.33) | <0.001^r^ | 1.37(1.13,1.68) | 0.002^r^ | 1.50(1.33,1.69) | <0.001^f^ |
| *XRCC1* | rs1799782 | 1.04(1.00,1.07) | 0.044^f^ | 1.05(0.99,1.11) | 0.085^f^ | 1.05(1.01,1.09) | 0.030^f^ | 1.03(1.00,1.05) | 0.032^f^ | 1.05(0.98,1.14) | 0.179^f^ |
| *XRCC1* | rs25487 | 1.35(1.04,1.74) | 0.025^r^ | 1.54(1.12,2.11) | 0.008^r^ | 1.69(1.05,2.70) | 0.030^r^ | 1.54(1.11,2.14) | 0.010^r^ | 1.37(0.91,2.05) | 0.131^r^ |
| **Related to metabolic pathway** | | | | | | | | | | | |
| *ALDH2* | rs671 | 1.01(0.94,1.10) | 0.762^f^ | 0.99(0.91,1.07) | 0.742^f^ | 1.10(0.87,1.39) | 0.444^f^ | 1.00(0.93,1.07) | 0.947^f^ | 1.11(0.87,1.42) | 0.391^f^ |
| *CYP1A1* | rs1048943 | 1.41(1.00,1.97) | 0.048^r^ | 1.26(0.88,1.81) | 0.205^r^ | 1.92(1.01,3.63) | 0.046^r^ | 1.43(0.95,2.15) | 0.088^r^ | 1.63(1.01,2.63) | 0.048^r^ |
| *CYP1A1* | rs4646903 | 1.05(0.97,1.15) | 0.237^f^ | 1.09(0.98,1.20) | 0.101^f^ | 1.10(0.92,1.31) | 0.295^f^ | 1.06(0.99,1.14) | 0.106^f^ | 1.02(0.83,1.26) | 0.824^f^ |
| *GC* | rs7041 | 1.04(0.93,1.16) | 0.490^f^ | 1.03(0.92,1.16) | 0.586^f^ | 1.10(0.82,1.47) | 0.529^f^ | 1.03(0.94,1.14) | 0.514^f^ | 1.07(0.79,1.45) | 0.671^f^ |
| *MTHFR* | rs1801131 | 1.00(0.98,1.02) | 0.993^f^ | 1.29(0.73,2.27) | 0.386^r^ | 1.01(0.99,1.03) | 0.374^f^ | 1.01(0.99,1.02) | 0.332^f^ | 0.99(0.94,1.04) | 0.679^f^ |
| *MTHFR* | rs1801133 | 1.04(0.91,1.18) | 0.582^r^ | 1.04(1.01,1.07) | 0.013^f^ | 1.11(0.89,1.38) | 0.375^r^ | 1.03(1.01,1.04) | 0.009^f^ | 0.99(0.80,1.23) | 0.932^r^ |
| *MTRR* | rs1801394 | 1.01(0.97,1.04) | 0.747^f^ | 0.99(0.95,1.04) | 0.661^f^ | 1.00(0.97,1.03) | 0.959^f^ | 1.00(0.98,1.02) | 0.827^f^ | 1.02(0.95,1.09) | 0.614^f^ |
| *NQO1* | rs1800566 | 1.25(1.18,1.32) | <0.001^f^ | 1.19(1.11,1.27) | <0.001^f^ | 1.53(1.36,1.72) | <0.001^f^ | 1.18(1.12,1.24) | <0.001^f^ | 1.47(1.28,1.69) | <0.001^f^ |
| *PNPLA3* | rs738409 | 1.65(1.00,2.71) | 0.049^r^ | 1.47(0.86,2.51) | 0.165^r^ | 2.67(1.09,6.55) | 0.032^r^ | 1.71(0.92,3.16) | 0.091^r^ | 2.15(1.14,4.06) | 0.019^r^ |
| **Related to signaling pathways and regulation of gene expression** | | | | | | | | | | | |
| *CCND1* | rs9344 | 1.07(0.87,1.31) | 0.537^r^ | 1.01(0.96,1.08) | 0.652^f^ | 1.15(0.73,1.79) | 0.547^r^ | 1.09(0.78,1.52) | 0.611^r^ | 1.06(0.93,1.20) | 0.404^f^ |
| *EGF* | rs4444903 | 1.05(1.02,1.08) | 0.001^f^ | 1.02(0.98,1.06) | 0.290^f^ | 1.05(1.01,1.08) | 0.006^f^ | 1.02(1.00,1.04) | 0.042^f^ | 1.11(1.04,1.18) | 0.001^f^ |

SNPs, single nucleotide polymorphisms; *OR*, odd ratios; *CI*, confidence intervals. ^f^, fixed model; ^r^, random model

Table S2 (Continued). Quantitative combination of genetic factors and HCC

| Gene | SNPs | Per-allele | | Heterozygous | | Homozygous | | Dominant model | | Recessive model | |
| --- | --- | --- | --- | --- | --- | --- | --- | --- | --- | --- | --- |
|  |  | *OR* (95%*CI*) | *P* | *OR* (95%*CI*) | *P* | *OR* (95%*CI*) | *P* | *OR* (95%*CI*) | *P* | *OR* (95%*CI*) | *P* |
| *ESR1* | rs2077647 | 1.71(0.80,3.65) | 0.168^r^ | 1.36(0.59,3.14) | 0.465^r^ | 2.67(0.66,10.81) | 0.167^r^ | 1.72(0.69,4.29) | 0.242^r^ | 2.22(0.72,6.89) | 0.167^r^ |
| *ESR1* | rs2234693 | 1.00(0.55,1.82) | 0.999^r^ | 0.93(0.85,1.02) | 0.105^f^ | 0.94(0.31,2.90) | 0.916^r^ | 0.84(0.43,1.64) | 0.601^r^ | 1.10(0.45,2.69) | 0.829^r^ |
| *ESR1* | rs9340799 | 1.24(1.06,1.46) | 0.007^f^ | 1.49(0.88,2.50) | 0.136^r^ | 1.62(0.97,2.70) | 0.067^f^ | 1.51(0.93,2.47) | 0.096^r^ | 1.43(0.85,2.42) | 0.178^f^ |
| *FAS* | rs1800682 | 1.17(0.91,1.51) | 0.213^r^ | 1.08(0.98,1.19) | 0.104^f^ | 1.36(0.80,2.33) | 0.258^r^ | 1.07(1.00,1.15) | 0.064^f^ | 1.21(0.72,2.04) | 0.467^r^ |
| *FGFR4* | rs351855 | 1.04(0.99,1.10) | 0.117^f^ | 1.03(0.97,1.09) | 0.358^f^ | 1.10(0.98,1.23) | 0.121^f^ | 1.03(0.99,1.07) | 0.194^f^ | 1.09(0.96,1.24) | 0.206^f^ |
| *KIF1B* | rs17401966 | 1.34(1.08,1.67) | 0.008^r^ | 1.30(0.87,1.94) | 0.205^r^ | 1.73(0.99,3.02) | 0.054^r^ | 1.53(0.93,2.54) | 0.096^r^ | 1.21(1.16,1.26) | <0.001^f^ |
| *miR-146a* | rs2910164 | 1.08(0.91,1.27) | 0.394^r^ | 1.07(1.04,1.11) | <0.001^f^ | 1.14(0.81,1.61) | 0.447^r^ | 1.18(0.98,1.41) | 0.077^r^ | 1.05(0.78,1.42) | 0.743^r^ |
| *miR-149* | rs2292832 | 1.13(0.99,1.28) | 0.066^r^ | 1.04(0.98,1.10) | 0.238^f^ | 1.30(1.04,1.61) | 0.019^r^ | 1.04(1.00,1.08) | 0.057^f^ | 1.12(1.01,1.25) | 0.038^f^ |
| *miR-196a2* | rs11614913 | 1.07(1.04,1.11) | <0.001^f^ | 1.18(0.99,1.41) | 0.059^r^ | 1.18(1.10,1.27) | <0.001^f^ | 1.22(1.06,1.41) | 0.006^r^ | 1.19(0.99,1.42) | 0.061^r^ |
| *miR-34b/c* | rs4938723 | 1.14(0.93,1.40) | 0.211^r^ | 1.12(0.91,1.38) | 0.281^r^ | 1.32(0.86,2.02) | 0.203^r^ | 1.16(0.91,1.48) | 0.239^r^ | 1.22(0.87,1.72) | 0.245^r^ |
| *miR-499* | rs3746444 | 1.11(1.00,1.23) | 0.050^f^ | 1.07(0.96,1.21) | 0.229^f^ | 1.27(0.96,1.69) | 0.096^f^ | 1.09(0.98,1.20) | 0.106^f^ | 1.24(0.93,1.66) | 0.145^f^ |
| *NFKBIA* | rs696 | 1.17(0.90,1.53) | 0.250^r^ | 1.20(0.91,1.57) | 0.191^r^ | 1.38(0.77,2.48) | 0.286^r^ | 1.23(0.90,1.67) | 0.188^r^ | 1.24(0.74,2.08) | 0.420^r^ |
| *STAT3* | rs2293152 | 1.08(1.03,1.13) | 0.002^f^ | 1.02(0.96,1.07) | 0.543^f^ | 1.17(1.06,1.28) | 0.002^f^ | 1.03(1.00,1.08) | 0.083^f^ | 1.22(1.09,1.37) | 0.001^f^ |
| *STAT4* | rs7574865 | 1.07(1.04,1.10) | <0.001^f^ | 1.06(1.02,1.10) | 0.005^f^ | 1.09(1.05,1.13) | <0.001^f^ | 1.04(1.02,1.06) | <0.001^f^ | 1.13(1.06,1.21) | <0.001^f^ |
| *TGF-β1* | rs1800469 | 1.27(1.04,1.56) | 0.022^r^ | 1.07(1.01,1.13) | 0.014^f^ | 1.61(1.06,2.46) | 0.026^r^ | 1.39(1.05,1.83) | 0.020^r^ | 1.31(0.98,1.76) | 0.064^r^ |

SNPs, single nucleotide polymorphisms; *OR*, odd ratios; *CI*, confidence intervals. ^f^, fixed model; ^r^, random model

Table S3. Heterogeneity and publication bias of included studies

| Factors | Heterogeneity | | Publication bias | | | |
| --- | --- | --- | --- | --- | --- | --- |
|  | *I*^2^ | *P* | Begger’s test | | Egger’s test | |
|  |  |  | *z* | *P* | *t* | *P* |
| **Non-genetic factors** |  |  |  |  |  |  |
| HBV infection | 97.60% | <0.001 | 0.63 | 0.530 | -0.10 | 0.917 |
| HCV infection | 92.40% | <0.001 | 1.07 | 0.286 | -5.24 | <0.001 |
| Smoking history | 92.30% | <0.001 | 3.32 | 0.001 | 1.36 | 0.174 |
| Drinking history | 93.90% | <0.001 | 6.24 | <0.001 | 7.97 | <0.001 |
| T2DM | 94.90% | <0.001 | 3.09 | 0.002 | -1.68 | 0.100 |
| Cirrhosis | 98.30% | <0.001 | 0.94 | 0.349 | -0.73 | 0.477 |
| Fatty liver | 96.60% | <0.001 | 0.09 | 0.928 | -3.41 | 0.003 |
| Family history of HBV | 84.20% | <0.001 | 0.07 | 0.945 | -1.14 | 0.281 |
| Family history of HCC | 83.70% | <0.001 | 0.64 | 0.522 | 2.44 | 0.053 |
| Family history of cancer | 88.20% | <0.001 | 2.08 | 0.038 | 0.10 | 0.925 |
| Aflatoxin-contaminated food | 78.50% | 0.001 | -0.24 | 1.000 | 0.49 | 0.655 |
| Fried and smoked food | 52.10% | 0.051 | 0.00 | 1.000 | -0.63 | 0.559 |
| **Genetic factors** |  |  |  |  |  |  |
| *COX-2* (rs5275) CT+CC | 0.00% | 0.605 | 0.00 | 1.000 | -0.81 | 0.567 |
| *COX-2* (rs689466) AG | 35.90% | 0.182 | -0.24 | 1.000 | 1.05 | 0.369 |
| *IL-6* (rs1800796) GC+CC | 0.00% | 0.475 | 0.73 | 0.462 | 1.15 | 0.333 |
| *IL-8* (rs4073) T | 44.70% | 0.143 | 0.34 | 0.734 | 0.08 | 0.940 |
| *IL-10* (rs1800872) CA+AA | 0.00% | 0.722 | 0.34 | 0.734 | 0.23 | 0.842 |
| *TNF-α* (rs1799964) TT | 16.90% | 0.307 | 0.34 | 0.734 | 0.40 | 0.727 |
| *hOGG1* (rs1052133) GC+GG | 13.30% | 0.316 | 0.00 | 1.000 | 0.13 | 0.918 |
| *XPC* (rs2228001) CA+CC | 42.50% | 0.157 | 0.34 | 0.734 | -0.75 | 0.532 |
| *GSTP1* (rs1695) AA | 0.00% | 0.488 | 1.02 | 0.308 | -6.29 | 0.024 |
| *MDM2* (rs2279744) GT+GG | 68.20% | <0.001 | 0.47 | 0.640 | 0.43 | 0.676 |
| *TP53* (rs1042522) GC | 44.20% | 0.056 | 1.40 | 0.161 | 1.39 | 0.197 |
| *XRCC1* (rs1799782) GA+AA | 0.00% | 0.507 | 1.13 | 0.260 | 3.35 | 0.029 |
| *XRCC1* (rs25487) CC | 74.70% | <0.001 | -0.10 | 1.000 | -2.62 | 0.034 |
| *MTHFR* (rs1801133) CT | 0.00% | 0.890 | 0.49 | 0.621 | -0.37 | 0.725 |
| *CYP1A1* (rs1048943) CT+CC | 62.90% | 0.029 | 0.73 | 0.462 | 1.56 | 0.216 |
| *NQO1* (rs1800566) TC | 0.00% | 0.647 | 0.24 | 0.806 | -0.15 | 0.889 |
| *PNPLA3* (rs738409) GC+GG | 82.40% | 0.001 | 0.34 | 0.734 | 1.23 | 0.344 |
| *EGF* (rs4444903) AA | 0.00% | 0.605 | 0.00 | 1.000 | -0.16 | 0.876 |

HBV, hepatitis B virus; HCV, hepatitis C virus; HCC, hepatocellular carcinoma; T2DM, type 2 diabetes mellitus. *I*^2^, *z,* and *t* are the test statistic of heterogeneity, Begger’s test, and Egger’s test;

^*^ *P* for heterogeneity; ^†^ *P* for Begger’s test; ^‡^ *P* for Egger’s test

Table S3 (Continued). Heterogeneity and publication bias of included studies

| Factors | Heterogeneity | | Publication bias | | | |
| --- | --- | --- | --- | --- | --- | --- |
|  | *I*^2^ | *P^*^* | Begger’s test | | Egger’s test | |
|  |  |  | *z* | *P*^†^ | *t* | *P*^‡^ |
| *KIF1B* (rs17401966) AG+AA | 50.70% | 0.087 | 1.71 | 0.086 | -1.47 | 0.239 |
| *miR-149* (rs2292832) TT | 25.20% | 0.253 | 1.71 | 0.086 | 1.66 | 0.196 |
| *miR-146a* (rs2910164) GC | 48.20% | 0.026 | 0.06 | 0.951 | 0.08 | 0.938 |
| *miR-196a2* (rs11614913) TT | 40.30% | 0.080 | 0.93 | 0.350 | 0.94 | 0.372 |
| *miR-499* (rs3746444) T | 0.00% | 0.813 | 0.73 | 0.462 | -1.05 | 0.371 |
| *STAT3* (rs2293152) GC+GG | 0.00% | 0.610 | 1.70 | 0.089 | 22.27 | 0.002 |
| *STAT4* (rs7574865) GT | 0.00% | 0.984 | -0.34 | 1.000 | -0.05 | 0.966 |
| *TGF-β1* (rs1800469) TC | 33.40% | 0.199 | 1.71 | 0.086 | 5.16 | 0.014 |

HBV, hepatitis B virus; HCV, hepatitis C virus; HCC, hepatocellular carcinoma; T2DM, type 2 diabetes mellitus. *I*^2^, *z,* and *t* are the test statistic of heterogeneity, Begger’s test, and Egger’s test;

^*^ *P* for heterogeneity; ^†^ *P* for Begger’s test; ^‡^ *P* for Egger’s test

Table S4. The level of evidence for genetic factors

| Gene | SNPs | Allele  （a/b） | Genotype of HCC | | | Genotype of control | | | *OR* (95%*CI*) | *P* | *FPRP* | | | Venice Criteria |
| --- | --- | --- | --- | --- | --- | --- | --- | --- | --- | --- | --- | --- | --- | --- |
|  |  |  | aa | ab | bb | aa | ab | bb |  |  | 0.15 | 0.1 | 0.01 |  |
| **Related to inflammation and immune response** | | | | | | | | | | | | | | |
| *COX-2* | rs5275 | C/T | 60 | 368 | 772 | 51 | 461 | 958 | 1.47(1.03,2.11) | 0.036 | 0.277 | 0.378 | 0.870 | BAB |
| *COX-2* | rs689466 | A/G | 491 | 857 | 388 | 566 | 1,117 | 553 | 1.19(1.01,1.39) | 0.034 | 0.138 | 0.203 | 0.737 | BBB |
| *IL-6* | rs1800796 | G/C | 120 | 780 | 1,472 | 106 | 739 | 1,559 | 1.08(1.00,1.17) | 0.046 | 0.252 | 0.349 | 0.855 | BAB |
| *IL-8* | rs4073 | A/T | 158 | 578 | 317 | 177 | 489 | 441 | 1.12(1.04,1.20) | 0.003 | 0.007 | 0.011 | 0.113 | BBB |
| *IL-10* | rs1800872 | C/A | 222 | 637 | 555 | 228 | 761 | 810 | 1.11(1.04,1.18) | 0.003 | 0.005 | 0.007 | 0.075 | BAB |
| *TNF-α* | rs1799964 | C/T | 88 | 496 | 937 | 82 | 769 | 1,427 | 1.10(1.00,1.20) | 0.045 | 0.153 | 0.223 | 0.759 | BAB |
| **Related to DNA synthesis and damage repair** | | | | | | | | | | | | | | |
| *GSTP1* | rs1695 | G/A | 90 | 307 | 365 | 68 | 348 | 433 | 1.35(1.02,1.80) | 0.039 | 0.233 | 0.325 | 0.841 | BAB |
| *hOGG1* | rs1052133 | G/C | 136 | 347 | 128 | 97 | 299 | 213 | 1.42(1.14,1.77) | 0.002 | 0.015 | 0.023 | 0.207 | BAB |
| *MDM2* | rs2279744 | G/T | 1,103 | 1,781 | 764 | 1,279 | 2,305 | 1,125 | 1.20(1.02,1.40) | 0.027 | 0.104 | 0.156 | 0.67 | ACA |
| *TP53* | rs1042522 | G/C | 1,374 | 2,788 | 1,479 | 1,953 | 4,075 | 2,306 | 1.17(1.02,1.36) | 0.030 | 0.188 | 0.269 | 0.802 | ABA |
| *XPC* | rs2228001 | C/A | 508 | 1,521 | 1,224 | 431 | 1,778 | 1,902 | 1.31(1.11,1.53) | 0.001 | 0.004 | 0.006 | 0.063 | BBB |
| *XRCC1* | rs1799782 | G/A | 643 | 461 | 94 | 640 | 485 | 130 | 1.04(1.00,1.07) | 0.044 | 0.037 | 0.058 | 0.405 | BAB |
| *XRCC1* | rs25487 | T/C | 260 | 1,199 | 1,257 | 213 | 1,211 | 2,345 | 1.35(1.04,1.74) | 0.025 | 0.128 | 0.189 | 0.719 | BCB |
| **Related to metabolic pathway** | | | | | | | | | | | | | | |
| *CYP1A1* | rs1048943 | C/T | 142 | 601 | 833 | 121 | 681 | 965 | 1.41(1.00,1.97) | 0.048 | 0.280 | 0.382 | 0.872 | BCB |

SNPs, single nucleotide polymorphisms; *OR*, odd ratios; *CI*, confidence intervals; FPRP, false positive reporting probability; Venice Criteria: this metric represents, in order, the total amount of evidence, reproducibility, and bias control

Table S4 (Continue). The level of evidence for genetic factors

| Gene | SNPs | Allele  （a/b） | Genotype of HCC | | | Genotype of control | | | *OR* (95%*CI*) | *P* | *FPRP* | | | Venice Criteria |
| --- | --- | --- | --- | --- | --- | --- | --- | --- | --- | --- | --- | --- | --- | --- |
|  |  |  | aa | ab | bb | aa | ab | bb |  |  | 0.15 | 0.1 | 0.01 |  |
| *MTHFR* | rs1801133 | C/T | 1,267 | 1,596 | 513 | 2,173 | 3,126 | 1,303 | 1.04(1.01,1.07) | 0.013 | 0.037 | 0.058 | 0.405 | AAA |
| *NQO1* | rs1800566 | T/C | 391 | 647 | 346 | 272 | 634 | 521 | 1.25(1.18,1.32) | <0.001 | <0.001 | <0.001 | <0.001 | BAB |
| *PNPLA3* | rs738409 | G/C | 168 | 413 | 341 | 313 | 1,176 | 1,142 | 1.65(1.00,2.71) | 0.049 | 0.435 | 0.550 | 0.931 | BCB |
| **Related to signaling pathways and regulation of gene expression** | | | | | | | | | | | | | | |
| *EGF* | rs4444903 | G/A | 917 | 717 | 149 | 1,229 | 1,148 | 265 | 1.05(1.02,1.08) | 0.001 | 0.004 | 0.006 | 0.064 | BAA |
| *KIF1B* | rs17401966 | A/G | 2,200 | 1,181 | 161 | 1,558 | 1,244 | 251 | 1.34(1.08,1.67) | 0.008 | 0.058 | 0.089 | 0.519 | BCA |
| *miR-146a* | rs2910164 | G/C | 860 | 2,241 | 1,415 | 892 | 2,688 | 2,028 | 1.07(1.04,1.11) | <0.001 | 0.002 | 0.003 | 0.029 | ABB |
| *miR-149* | rs2292832 | C/T | 458 | 914 | 709 | 530 | 1,085 | 842 | 1.30(1.04,1.61) | 0.019 | 0.092 | 0.139 | 0.639 | BBB |
| *miR-196a2* | rs11614913 | C/T | 915 | 2,084 | 1,271 | 982 | 2,659 | 1,771 | 1.07(1.04,1.11) | <0.001 | 0.002 | 0.003 | 0.029 | ABB |
| *miR-499* | rs3746444 | C/T | 83 | 335 | 662 | 92 | 463 | 1,108 | 1.11(1.00,1.23) | 0.050 | 0.208 | 0.294 | 0.821 | BAB |
| *STAT3* | rs2293152 | G/C | 423 | 802 | 450 | 540 | 1,299 | 748 | 1.08(1.03,1.13) | 0.002 | 0.005 | 0.008 | 0.078 | BAA |
| *STAT4* | rs7574865 | G/T | 717 | 597 | 105 | 1,832 | 1,841 | 464 | 1.07(1.04,1.10) | <0.001 | <0.001 | <0.001 | <0.001 | BAB |
| *TGF-β1* | rs1800469 | T/C | 413 | 880 | 521 | 524 | 1,223 | 805 | 1.27(1.04,1.56) | 0.022 | 0.120 | 0.178 | 0.705 | BBB |

SNPs, single nucleotide polymorphisms; *OR*, odd ratios; *CI*, confidence intervals; FPRP, false positive reporting probability; Venice Criteria: this metric represents, in order, the total amount of evidence, reproducibility, and bias control

Table S5. Comparison of AUC, AIC, BIC, NRI and IDI between different risk prediction model in patients with HBV infection or cirrhosis

| Groups | AUC (95%*CI*) | Delong *P* | AIC | BIC | NRI (95%*CI*) | IDI (95%*CI*) |
| --- | --- | --- | --- | --- | --- | --- |
| **HBV infection** |  | <0.001 |  |  |  |  |
| PRS | 0.65(0.59,0.70) |  | 957.76 | 967.02 | - | - |
| PRS+environmental factors | 0.77(0.73,0.80) |  | 818.57 | 841.71 | 0.38(0.29,0.47)^*^ | 0.18(0.15,0.21)^*^ |
| **Cirrhosis** |  | 0.614 |  |  |  |  |
| PRS | 0.65(0.60,0.71) |  | 604.49 | 613.36 | - | - |
| PRS+environmental factors | 0.65(0.59,0.70) |  | 545.37 | 567.55 | 0.15(0.07,0.23)^*^ | 0.08(0.06,0.10)^*^ |

^*^*P* < 0.05, AUC, area under curve; CI, confidence intervals; AIC, Akaike information criterion; BIC, Bayesian information criterion; NRI, net reclassification improvement; IDI, integrated discrimination improvement; PRS, polygenic risk score; environmental factors: drinking history, HBV infection, cirrhosis and family history of HCC

Figure S1. Association analysis of SNPs in genes related to inflammation and immune response with the risk of HCC

Adjusted for drinking history, T2DM, family history of HCC, HBV infection, and cirrhosis. HCC, hepatocellular carcinoma; *OR*, odd ratios; *CI*, confidence intervals; SNPs, single nucleotide polymorphisms

Figure S2. Association analysis of SNPs in genes related to DNA synthesis and damage repair with the risk of HCC

Adjusted for drinking history, T2DM, family history of HCC, HBV infection, and cirrhosis. HCC, hepatocellular carcinoma; *OR*, odd ratios; *CI*, confidence intervals; SNPs, single nucleotide polymorphisms

Figure S3. Association analysis of SNPs in genes related to metabolic pathway with the risk of HCC Adjusted for drinking history, T2DM, family history of HCC, HBV infection, and cirrhosis. HCC, hepatocellular carcinoma; *OR*, odd ratios; *CI*, confidence intervals; SNPs, single nucleotide polymorphisms

Figure S4. Association analysis of SNPs in genes related to signaling pathways and regulation of gene expression with the risk of HCC

Adjusted for drinking history, T2DM, family history of HCC, HBV infection, and cirrhosis. HCC, hepatocellular carcinoma; *OR*, odd ratios; *CI*, confidence intervals; SNPs, single nucleotide polymorphisms


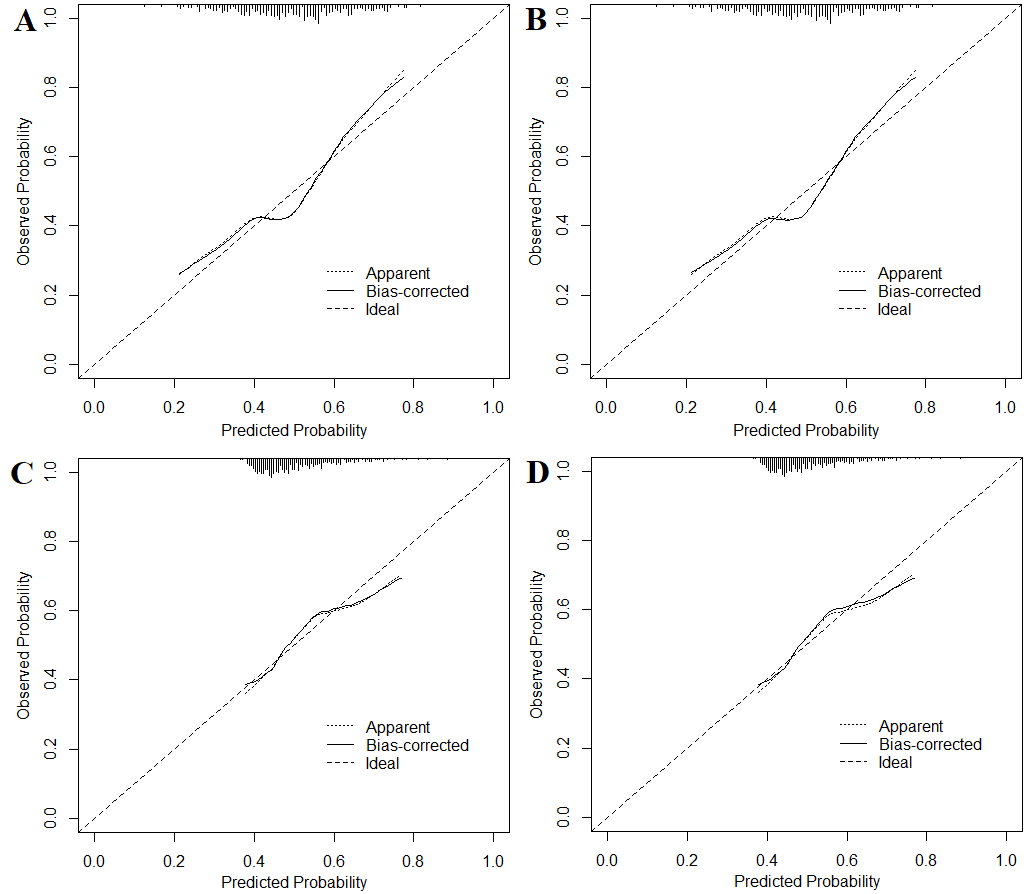


Figure S5. Calibration curves of wGRS and PRS

(A) PRS in training set; (B) PRS in validation set; (C) wGRS in training set; (D) wGRS in validation set
